# Supplementary material for: A plasma-based DNA test for quantification of disease burden in acute myeloid leukemia patients undergoing bone marrow transplantation
Source: Proc Natl Acad Sci U S A. 2026 Apr 14;123(16):e2537987123. doi: 10.1073/pnas.2537987123 (PMC13099560; doi:10.1073/pnas.2537987123)
Supplement: Supplementary file 1 — Appendix 01 (PDF) [file pnas.2537987123.sapp.pdf]

PNAS

Supporting Information for

A plasma-based DNA test for quantification of disease burden in acute myeloid leukemia patients undergoing bone marrow transplantation

Y. Wang et al.

This PDF file includes:

Figures S1 to S2
Tables S1 to S4

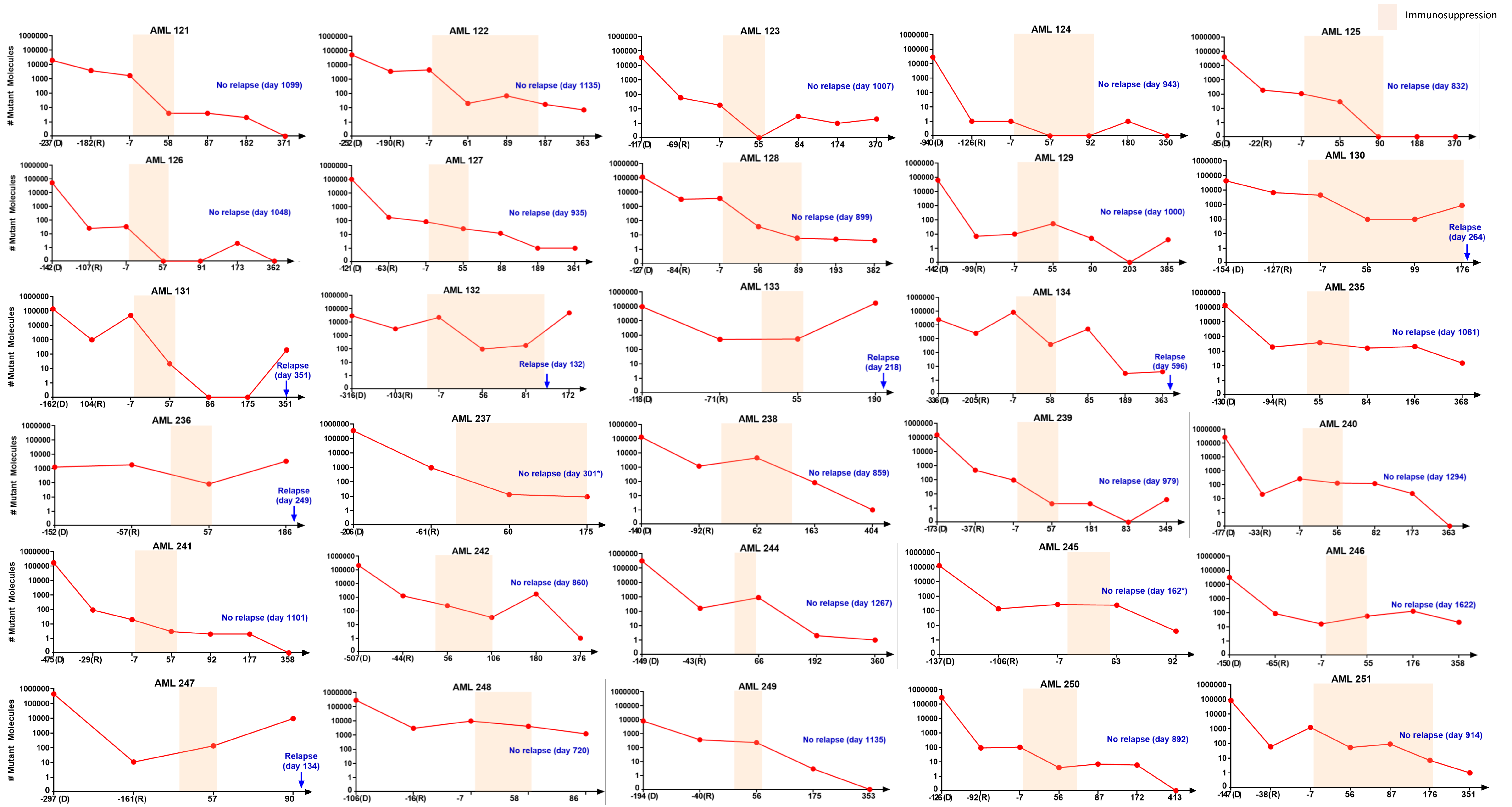

Figure S1: Number of mutant molecules before and after transplantation. Plots of the number of mutant molecules detected at diagnosis (D), complete remission (R), and at other time points for all 30 patients enrolled in this study. For each patient, 150 ng of bone marrow or peripheral blood cell DNA (see SI Table S1) was assayed by v96 for timepoints D and R, while cell-free DNA from 10-mL of plasma was assayed by v96 for the other time points. The x-axis represents the days relative to transplantation for these timepoints, with transplantation performed at day 0. For patients who relapsed, the post-transplantation day of relapse is indicated. For patients without relapse, the post-transplantation day of last follow-up is indicated (*Patients AML 237 and AML 245 passed away from unrelated causes without evidence of leukemic relapse). Colored regions represent intervals during which immunosuppression was administered.

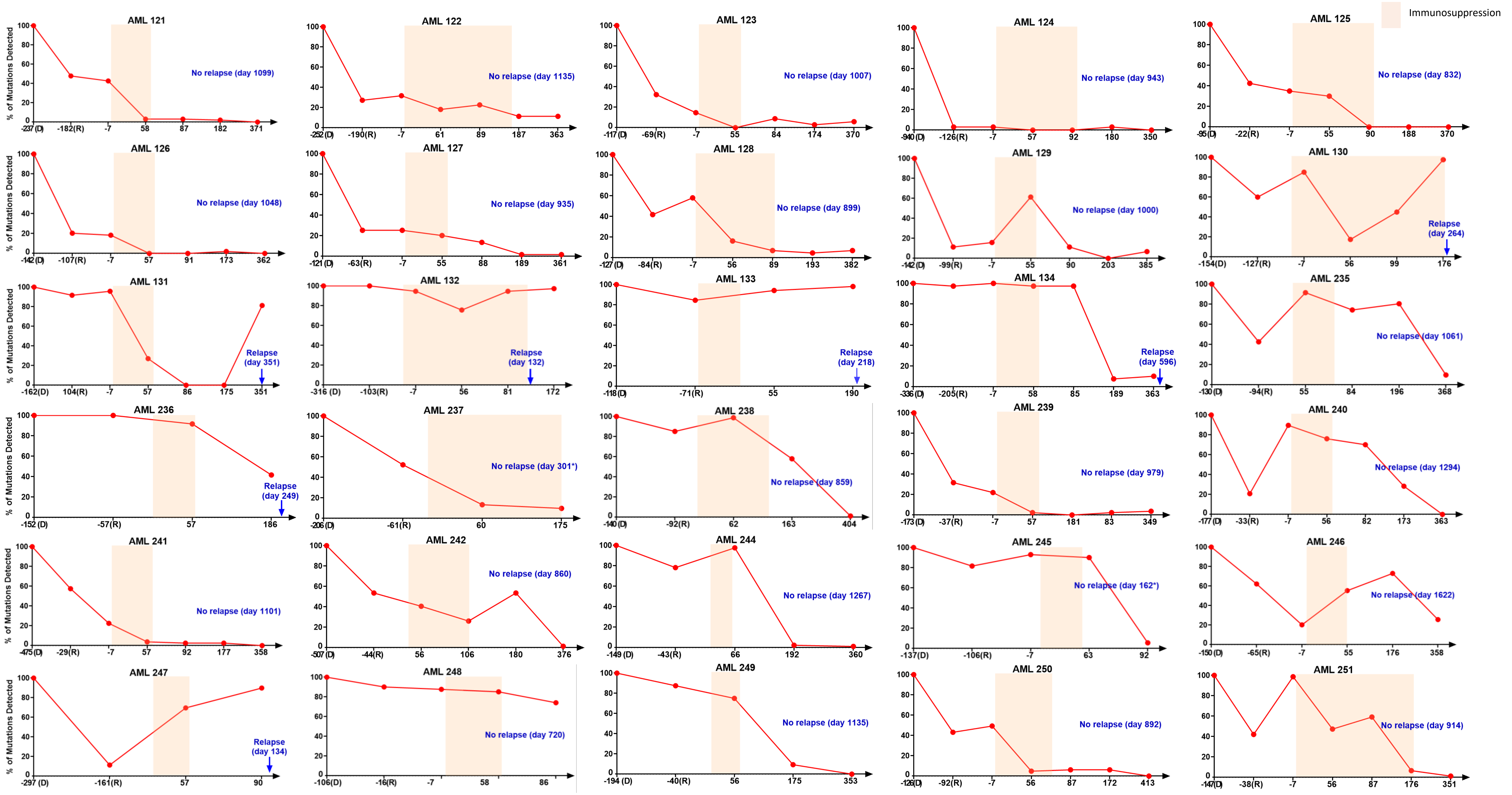

Figure S2: Fraction of distinct mutations (rather than mutant molecules) detected before and after transplantation. Plots of the fraction of distinct mutations detected at complete remission (R) and at other time points, relative to the total number of distinct mutations detected at diagnosis (D). This is different from the total number of mutant molecules depicted in Figure S1. The fractions in Figure S2 were normalized to the number of distinct mutations detected at diagnosis (always 100%). For example, if a patient had 80 distinct mutations detected at diagnosis, and 40 of those mutations were detected at remission, the fraction on the y-axis for the remission sample would be 50%. Bone marrow or peripheral blood cell DNA (see SI Table S2) was assayed by v96 for timepoints D and R, while cell-free DNA from plasma was assayed by v96 for the other time points. The x-axis represents the days relative to transplantation for these timepoints, with transplantation performed at day 0. For patients who relapsed, the post-transplantation day of relapse is indicated. For patients without relapse, the post-transplantation day of last follow-up is indicated (*Patients AML 237 and AML 245 passed away from unrelated causes without evidence of leukemic relapse). Colored regions represent intervals during which immunosuppression was administered.

Table S1: Patient characteristics.

Patient ID	*Age*	*Gender*	*Diagnosis*	*Cytogenetics*	*Induction therapy*	*Consolidation therapy*	*Fraction blasts by flow cytometry at remission*	*Match status (10/10, 9/10, 8/10, etc).*	*Conditioning*	*Type of transplant*	*Relapse Status*	*Immunsuppression end (Days post-alloHCT)*	*Time of relapse or last follow-up (Days post-alloHCT)*	*Decrease in mutation burden after Day 60*	*Decrease in mutation burden after immunosuppression discontinued*	*Post-transplant therapy*
AML 121	69	Male	AML	Normal	HMA + venetoclax	HMA + venetoclax	negative	Unrelated (10/10)	14.5 mg/kg day -6, -5 cyclophosphamide, 30mg/m2, day -6 to day -2 fludarabine, and TBI 400cGy	non-myeloablative fully matched unrelated donor peripheral blood stem cell transplant	No relapse	60	1099	Yes	Yes	None
AML 122	62	Female	AML	Normal	7+3+GO	HiDAC x2	negative	Unrelated (8/10)	14.5 mg/kg day -6, -5 cyclophosphamide, 30mg/m2, day -6 to day -2 fludarabine, and TBI 400cGy	non-myeloablative mismatched unrelated donor peripheral blood stem cell transplant	No relapse	173	1135	Yes	Yes	ivosidenib
AML 123	36	Female	AML	Normal	7+3+ midostaurin	HiDAC	negative	Haploidentical (5/10)	14.5 mg/kg day -6, -5 cyclophosphamide, 30mg/m2, day -6 to day -2 fludarabine, and TBI 400cGy	non-myeloablative haploidentical bone marrow transplant	No relapse	60	1007	No mutation detected at Day 60	No mutations detected prior to immunosupression cessation	gilteritinib
AML 124	37	Female	rAML	inv(16)(p13.1q22)	7+3+GO	HiDAC	negative	Unrelated (9/10)	14.5 mg/kg day -6, -5 cyclophosphamide, 30mg/m2, day -6 to day -2 fludarabine, and TBI 200cGy	non-myeloablative mismatched unrelated donor peripheral blood stem cell transplant	No relapse	92	943	No mutation detected at Day 60	No mutations detected prior to immunosupression cessation	None
AML 125	68	Male	AML	Normal	7+3	none	negative	Haploidentical (5/10)	14.5 mg/kg day -6, -5 cyclophosphamide, 30mg/m2, day -6 to day -2 fludarabine, and TBI 400cGy	non-myeloablative haploidentical bone marrow transplant	No relapse	107	832	Yes	No mutations detected prior to immunosupression cessation	enasidenib
AML 126	51	Female	AML	Normal	7+3	HiDAC x2	negative	Haploidentical (5/10)	14.5 mg/kg day -6, -5 cyclophosphamide, 30mg/m2, day -6 to day -2 fludarabine, and TBI 400cGy	non-myeloablative haploidentical bone marrow transplant	No relapse	60	1048	No mutation detected at Day 60	No mutations detected prior to immunosupression cessation	None
AML 127	70	Male	AML	del(13)(q12q14)	Vyxeos+ midostaurin	Vyxeos + midostaurin	negative	Haploidentical (5/10)	14.5 mg/kg day -6, -5 cyclophosphamide, 30mg/m2, day -6 to day -2 fludarabine, and TBI 400cGy	non-myeloablative haploidentical bone marrow transplant	No relapse	60	935	Yes	Yes	gilteritinib
AML 128	75	Female	AML	Normal	7+3+ midostaurin	HiDAC + gilteritinib	negative	Haploidentical (8/10)	14.5 mg/kg day -6, -5 cyclophosphamide, 30mg/m2, day -6 to day -2 fludarabine, and TBI 400cGy	non-myeloablative haploidentical bone marrow transplant	No relapse	100	899	Yes	Yes	gilteritinib, ivosidenib
AML 129	51	Male	AML	Normal	7+3+ midostaurin	HiDAC + gilteritinib	negative	Unrelated (10/10)	1 mg/kg/dose, Oral, Every 6 hours Busulphan day -8 and -7 and 1 mg/kg/dose, Oral, Every 5 hours Busulphan on day -6. 50mg/kg/dose cyclophospahmide on days -4 and -3	myeloablative allogeneic transplant fully matched unrelated donor peripheral blood stem cell transplant	No relapse	60	1000	Yes	Yes	gilteritinib
AML 130	74	Male	AML	Complex	HMA + venetoclax	HMA + venetoclax	negative	Unrelated (8/10)	14.5 mg/kg day -6, -5 cyclophosphamide, 30mg/m2, day -6 to day -2 fludarabine, and TBI 400cGy	non-myeloablative mismatched unrelated donor peripheral blood stem cell transplant	Relapsed	180	264	No	Immunosupression stopped after last time point	None
AML 131	67	Male	sAML	Complex	Vyxeos	Vyxeos	0.8%	Haploidentical (5/10)	14.5 mg/kg day -6, -5 cyclophosphamide, 30mg/m2, day -6 to day -2 fludarabine, and TBI 400cGy	non-myeloablative haploidentical peripheral blood stem cell transplant	Relapsed	60	351	Yes	Yes	Donor lymphocyte infusion
AML 132	66	Female	AML-TP53	Complex	Decitabine	none	equivocal	Haploidentical (5/10)	14.5 mg/kg day -6, -5 cyclophosphamide, 30mg/m2, day -6 to day -2 fludarabine, and TBI 400cGy	non-myeloablative haploidentical peripheral blood stem cell transplant	Relapsed	130	132	No	No	None
AML 133	63	Male	AML-MRC	Normal	7+3+ midostaurin	HiDAC + midostaurin	negative	Haploidentical (5/10)	14.5 mg/kg day -6, -5 cyclophosphamide, 30mg/m2, day -6 to day -2 fludarabine, and TBI 400cGy	non-myeloablative haploidentical peripheral blood stem cell transplant	Relapsed	71	218	No	No	None
AML 134	64	Male	sAML	Complex	Decitabine+ ruxolitinib	none	0.8%	Haploidentical (5/10)	14.5 mg/kg day -6, -5 cyclophosphamide, 30mg/m2, day -6 to day -2 fludarabine, and TBI 400cGy	non-myeloablative haploidentical peripheral blood stem cell transplant	Relapsed	60	596	Yes	Yes	None
AML 235	51	Male	AML	-Y,t(8;21)(q22;q22)	7+3+GO	HiDAC	negative	Haploidentical(5/10)	14.5 mg/kg day -6, -5 cyclophosphamide, 30mg/m2,day -6 to day -2 fludarabine, and TBI 400cGy	non-myeloablative haploidentical bone marrow transplant	No relapse	60	1061	Yes	Yes	None
AML 236	61	Male	MDS	Complex	Decitabine + venetoclax	Decitabine + venetoclax	0.08%	Haploidentical(5/10)	14.5 mg/kg day -6, -5 cyclophosphamide, 30mg/m2,day -6 to day -2 fludarabine, and TBI 400cGy	non-myeloablative haploidentical peripheral blood stem cell transplant	Relapsed	60	249	No	No	aza,Decitabine
AML 237	71	Female	AML	Normal	Vyxeo	HiDAC	negative	Unrelated (7/10)	14.5 mg/kg day -6, -5 cyclophosphamide, 30mg/m2,day -6 to day -2 fludarabine, and TBI 400cGy	non-myeloablative mismatched unrelated donor peripheral blood stem cell transplant	No relapse	180	301	Yes	Immunosupression stopped after last time point	None
AML 238	66	Female	AML	Normal	Vyxeos	HiDAC	negative	Unrelated (10/10)	14.5 mg/kg day -6, -5 cyclophosphamide, 30mg/m2,day -6 to day -2 fludarabine, and TBI 400cGy	non-myeloablative fully matched unrelated donor peripheral blood stem cell transplant	No relapse	138	859	Yes	Yes	None
AML 239	63	Female	AML	inv(9)(p12q13)	7+3+ midostaurin	HiDAC + gilteritinib	negative	Unrelated (9/10)	14.5 mg/kg day -6, -5 cyclophosphamide, 30mg/m2,day -6 to day -2 fludarabine, and TBI 200cGy	non-myeloablative mismatched unrelated donor peripheral blood stem cell transplant	No relapse	90	979	Yes	Yes	gilteritinib
AML 240	64	Male	AML	Normal	Decitabine + venetoclax	Venetoclax	negative	Haploidentical(5/10)	14.5 mg/kg day -6, -5 cyclophosphamide, 30mg/m2,day -6 to day -2 fludarabine, and TBI 400cGy	non-myeloablative haploidentical peripheral blood stem cell transplant	No relapse	57	1294	Yes	Yes	None
AML 241	58	Male	AML	insufficient cell growth	7+3	Decitabine + venetoclax	0.5%	Haploidentical(5/10)	14.5 mg/kg day -6, -5 cyclophosphamide, 30mg/m2,day -6 to day -2 fludarabine, and TBI 400cGy	non-myeloablative haploidentical peripheral blood stem cell transplant	No relapse	60	1101	Yes	Yes	None

AML 242	69	Male	AML	+8	Azacitidine + venetoclax	Azacitidine + venetoclax	negative	Unrelated (8/10)	14.5 mg/kg day -6, -5 cyclophosphamide, 30mg/m2,day -6 to day -2 fludarabine, and TBI 400cGy	non-myeloablative mismatched unrelated donor peripheral blood stem cell transplant	No relapse	106	860	Yes	Yes	None
AML 244	50	Female	AML	Normal	7+3+ midostaurin	HiDAC + midostaurin	negative	Haploidentical(5/10)	14.5 mg/kg day -6, -5 cyclophosphamide, 30mg/m2,day -6 to day -2 fludarabine, and TBI 400cGy	non-myeloablative haploidentical bone marrow transplant	No relapse	60	1267	Yes	Yes	None
AML 245	66	Male	AML	Normal	7+3+ midostaurin	HiDAC + midostaurin	negative	Haploidentical(5/10)	14.5 mg/kg day -6, -5 cyclophosphamide, 30mg/m2,day -6 to day -2 fludarabine, and TBI 400cGy	non-myeloablative haploidentical bone marrow transplant	No relapse	57	162	Yes	Yes	None
AML 246	57	Male	MDS	Complex	Decitabine	Decitabine	negative	Haploidentical(5/10)	14.5 mg/kg day -6, -5 cyclophosphamide, 30mg/m2,day -6 to day -2 fludarabine, and TBI 400cGy	non-myeloablative haploidentical peripheral blood stem cell transplant	No relapse	60	1622	Yes	Yes	None
AML 247	35	Female	AML	t(6:11)(q27;q23)	7+3+GO	HiDAC	negative	Haploidentical (7/10)	14.5 mg/kg day -6, -5 cyclophosphamide, 30mg/m2,day -6 to day -2 fludarabine, and TBI 400cGy	non-myeloablative haploidentical peripheral blood stem cell transplant	Relapsed	60	134	No	No	None
AML 248	59	Male	AML	Normal	Vyxeos	HiDAC	negative	Haploidentical(5/10)	14.5 mg/kg day -6, -5 cyclophosphamide, 30mg/m2,day -6 to day -2 fludarabine, and TBI 400cGy	non-myeloablative haploidentical bone marrow transplant	No relapse	60	720	Yes	Yes	enasidenib
AML 249	71	Male	AML	Complex	Vyxeos	Vyxeos	0.8%	Haploidentical (5/10)	14.5 mg/kg day -6, -5 cyclophosphamide, 30mg/m2,day -6 to day -2 fludarabine, and TBI 400cGy	non-myeloablative haploidentical peripheral blood stem cell transplant	No relapse	66	1135	Yes	Yes	None
AML 250	54	Female	AML	Normal	7+3+ midostaurin	HiDAC	negative	Haploidentical(5/10)	14.5 mg/kg day -6, -5 cyclophosphamide, 30mg/m2,day -6 to day -2 fludarabine, and TBI 400cGy	non-myeloablative haploidentical bone marrow transplant	No relapse	70	892	Yes	Yes	gilteritinib
AML 251	62	Female	AML	Complex	7+3	Azacitidine + venetoclax	<0.1%	Unrelated (9/10)	14.5 mg/kg day -6, -5 cyclophosphamide, 30mg/m2,day -6 to day -2 fludarabine, and TBI 400cGy	non-myeloablative mismatched unrelated donor peripheral blood stem cell transplant	No relapse	180	914	Yes	Yes	None

sAML = secondary AML, rAML = relapsed AML, AML-TP53 = TP53-mutated AML; AML-MRC = Acute Myeloid Leukemia with myelodysplasia-related changes; HMA = hypomethylating agent; GO = gemtuzumab ozogamicin, HiDAC = High-Dose Cytarabine

Table S2: Numbers of mutants detected and mutant allele frequencies in the mutations assessed through v96 as well as the driver gene mutations assessessed in the same DNA samples. For each patient, 150 ng of bone marrow or peripheral blood cell DNA was assayed, and all the DNA purified from 10-mL of plasma was assayed.

Patient	Sample Type	Time point	Day post-alloHCT	*Day on which immunsuppression ended (Days post-alloHCT)*	*Total umber of v96 mutations assessed*	*Number of v96 mutations found*	*Fraction of v96 mutations found*	*Total number of mutant molecules found in v96*	*Average number of molecules assessed in v96 (mutant + non-mutant)*	*Average Mutant Allele Frequency in v96*	*Total number of driver gene mutations assessed*	*Number of positive driver gene mutations in sample*	*Total number of mutant molecules in driver genes*	*Average number of molecules assessed in driver genes (mutant + non-mutant)*	*Average mutant allele fraction in driver genes*
AML 121	Bone Marrow	Diagnostic	Minus 237	60	96	96	100.0%	19672	427618	4.60%	1	1	31	583	5.32%
AML 121	Bone Marrow	Remission	Minus 182	60	96	46	47.9%	3752	570555	0.66%	1	0	0	838	0.00%
AML 121	Plasma	0 month	Minus 7	60	96	41	42.7%	1664	399406	0.42%	1	0	0	329	0.00%
AML 121	Plasma	2 months	58	60	96	3	3.1%	4	826985	0.00%	1	0	0	597	0.00%
AML 121	Plasma	3 months	87	60	96	3	3.1%	4	889803	0.00%	1	0	0	432	0.00%
AML 121	Plasma	6 months	182	60	96	2	2.1%	2	455120	0.00%	1	0	0	583	0.00%
AML 121	Plasma	12 months	371	60	96	0	0.0%	0	459369	0.00%	1	0	0	748	0.00%
AML 122	Peripheral blood	Diagnostic	Minus 252	173	44	44	100.0%	50290	109924	45.75%	3	3	3025	10709	28.25%
AML 122	Peripheral blood	Remission	Minus 190	173	44	12	27.3%	3546	235788	1.50%	3	0	0	22960	0.00%
AML 122	Plasma	0 month	Minus 7	173	44	14	31.8%	4503	237439	1.90%	3	0	0	24150	0.00%
AML 122	Plasma	2 months	61	173	44	8	18.2%	20	210861	0.01%	3	0	0	24201	0.00%
AML 122	Plasma	3 months	89	173	44	10	22.7%	69	491933	0.01%	3	0	0	47374	0.00%
AML 122	Plasma	6 months	187	173	44	5	11.4%	17	227614	0.01%	3	0	0	23047	0.00%
AML 122	Plasma	12 months	363	173	44	5	11.4%	7	197021	0.00%	3	0	0	17861	0.00%
AML 123	Peripheral blood	Diagnostic	Minus 117	60	34	34	100.0%	35926	82759	43.41%	3	3	2280	5561	41.00%
AML 123	Peripheral blood	Remission	Minus 69	60	34	11	32.4%	59	137030	0.04%	3	1	3	10446	0.03%
AML 123	Plasma	0 month	Minus 7	60	34	5	14.7%	18	46908	0.04%	3	0	0	2803	0.00%
AML 123	Plasma	2 months	55	60	34	0	0.0%	0	59172	0.00%	3	0	0	4650	0.00%
AML 123	Plasma	3 months	84	60	34	3	8.8%	3	142714	0.00%	3	0	0	8631	0.00%
AML 123	Plasma	6 months	174	60	34	1	2.9%	1	138898	0.00%	3	0	0	8410	0.00%
AML 123	Plasma	12 months	370	60	34	2	5.9%	2	59217	0.00%	3	0	0	3558	0.00%
AML 124	Bone Marrow	Diagnostic	Minus 940	92	33	33	100.0%	28684	72941	39.32%	3	3	721	10805	6.67%
AML 124	Bone Marrow	Remission	Minus 126	92	33	1	3.0%	1	134353	0.00%	3	0	0	18659	0.00%
AML 124	Plasma	0 month	Minus 7	92	33	1	3.0%	1	56937	0.00%	3	0	0	6499	0.00%
AML 124	Plasma	2 months	57	92	33	0	0.0%	0	84440	0.00%	3	0	0	11624	0.00%
AML 124	Plasma	3 months	92	92	33	0	0.0%	0	134460	0.00%	3	0	0	18492	0.00%
AML 124	Plasma	6 months	180	92	33	1	3.0%	1	64284	0.00%	3	0	0	7783	0.00%
AML 124	Plasma	12 months	350	92	33	0	0.0%	0	84200	0.00%	3	0	0	9742	0.00%
AML 125	Peripheral blood	Diagnostic	Minus 95	107	40	40	100.0%	41019	109490	37.46%	1	1	347	1641	21.15%
AML 125	Peripheral blood	Remission	Minus 22	107	40	17	42.5%	188	208326	0.09%	1	0	0	3096	0.00%
AML 125	Plasma	0 month	Minus 7	107	40	14	35.0%	107	124905	0.09%	1	0	0	758	0.00%
AML 125	Plasma	2 months	55	107	40	12	30.0%	29	441348	0.01%	1	0	0	1204	0.00%
AML 125	Plasma	3 months	90	107	40	0	0.0%	0	247780	0.00%	1	0	0	689	0.00%
AML 125	Plasma	6 months	188	107	40	0	0.0%	0	215457	0.00%	1	0	0	562	0.00%
AML 125	Plasma	12 months	370	107	40	0	0.0%	0	75175	0.00%	1	0	0	745	0.00%
AML 126	Bone Marrow	Diagnostic	Minus 142	60	49	49	100.0%	53414	141502	37.75%	1	1	1978	4844	40.83%
AML 126	Bone Marrow	Remission	Minus 107	60	49	10	20.4%	25	283381	0.01%	1	1	3	8577	0.03%
AML 126	Plasma	0 month	Minus 7	60	49	9	18.4%	33	284688	0.01%	1	1	4	10797	0.04%
AML 126	Plasma	2 months	57	60	49	0	0.0%	0	202496	0.00%	1	0	0	7760	0.00%
AML 126	Plasma	3 months	91	60	49	0	0.0%	0	236950	0.00%	1	0	0	9540	0.00%
AML 126	Plasma	6 months	173	60	49	1	2.0%	2	430344	0.00%	1	1	2	17939	0.01%
AML 126	Plasma	12 months	362	60	49	0	0.0%	0	263317	0.00%	1	0	0	9818	0.00%
AML 127	Peripheral blood	Diagnostic	Minus 121	60	59	59	100.0%	102151	227495	44.90%	2	2	448	4981	8.99%
AML 127	Peripheral blood	Remission	Minus 63	60	59	15	25.4%	173	380831	0.05%	2	0	0	6520	0.00%
AML 127	Plasma	0 month	Minus 7	60	59	15	25.4%	83	161768	0.05%	2	0	0	2648	0.00%
AML 127	Plasma	2 months	55	60	59	12	20.3%	26	697824	0.00%	2	0	0	8906	0.00%
AML 127	Plasma	3 months	88	60	59	8	13.6%	12	713661	0.00%	2	0	0	8525	0.00%
AML 127	Plasma	6 months	189	60	59	1	1.7%	1	785223	0.00%	2	0	0	11039	0.00%
AML 127	Plasma	12 months	361	60	59	1	1.7%	1	726358	0.00%	2	0	0	9438	0.00%
AML 128	Peripheral blood	Diagnostic	Minus 127	100	43	43	100.0%	112872	235202	47.99%	3	3	6684	15854	42.16%
AML 128	Peripheral blood	Remission	Minus 84	100	43	18	41.9%	3168	519903	0.61%	3	2	450	38367	1.17%
AML 128	Plasma	0 month	Minus 7	100	43	25	58.1%	3691	572531	0.64%	3	2	398	30114	1.32%
AML 128	Plasma	2 months	56	100	43	7	16.3%	38	493083	0.01%	3	2	9	27898	0.03%
AML 128	Plasma	3 months	89	100	43	3	7.0%	6	283016	0.00%	3	1	2	17322	0.01%
AML 128	Plasma	6 months	193	100	43	2	4.7%	5	257615	0.00%	3	1	1	16064	0.01%
AML 128	Plasma	12 months	382	100	43	3	7.0%	4	257526	0.00%	3	1	1	15564	0.01%
AML 129	Bone Marrow	Diagnostic	Minus 142	60	44	44	100.0%	63803	155167	41.12%	2	2	1670	5481	30.47%
AML 129	Bone Marrow	Remission	Minus 99	60	44	5	11.4%	7	305357	0.00%	2	0	0	10651	0.00%

AML 129	Plasma	0 month	Minus 7	60	44	7	15.9%	10	262417	0.00%	2	0	0	8635	0.00%
AML 129	Plasma	2 months	55	60	44	27	61.4%	54	550703	0.01%	2	0	0	20185	0.00%
AML 129	Plasma	3 months	90	60	44	5	11.4%	5	567902	0.00%	2	0	0	20299	0.00%
AML 129	Plasma	6 months	203	60	44	0	0.0%	0	303532	0.00%	2	0	0	10302	0.00%
AML 129	Plasma	12 months	385	60	44	3	6.8%	4	423185	0.00%	2	0	0	16063	0.00%
AML 130	Bone Marrow	Diagnostic	Minus 154	180	40	40	100.0%	43354	130268	33.28%	1	1	1101	3788	29.07%
AML 130	Bone Marrow	Remission	Minus 127	180	40	24	60.0%	6756	370843	1.82%	1	0	0	14528	0.00%
AML 130	Plasma	0 month	Minus 7	180	40	34	85.0%	4462	246264	1.81%	1	1	1	7039	0.01%
AML 130	Plasma	2 months	56	180	40	7	17.5%	96	629914	0.02%	1	0	0	17638	0.00%
AML 130	Plasma	3 months	99	180	40	18	45.0%	98	549073	0.02%	1	0	0	15528	0.00%
AML 130	Plasma	6 months	176	180	40	39	97.5%	874	776320	0.11%	1	1	9	19040	0.05%
AML 131	Peripheral blood	Diagnostic	Minus 162	60	48	48	100.0%	140365	293596	47.81%	1	1	7238	7541	95.98%
AML 131	Peripheral blood	Remission	Minus 104	60	48	44	91.7%	990	213964	0.46%	1	1	32	4604	0.70%
AML 131	Plasma	0 month	Minus 7	60	48	46	95.8%	50086	269543	18.58%	1	1	2346	6354	36.92%
AML 131	Plasma	2 months	57	60	48	13	27.1%	21	531339	0.00%	1	1	3	14469	0.02%
AML 131	Plasma	3 months	86	60	48	0	0.0%	0	201164	0.00%	1	1	1	4997	0.02%
AML 131	Plasma	6 months	175	60	48	0	0.0%	0	164554	0.00%	1	0	0	3901	0.00%
AML 131	Plasma	12 months	351	60	48	39	81.3%	194	166008	0.12%	1	1	10	3874	0.26%
AML 132	Bone Marrow	Diagnostic	Minus 316	130	37	37	100.0%	30016	113008	26.56%	1	1	1261	5011	25.16%
AML 132	Bone Marrow	Remission	Minus 103	130	37	37	100.0%	3119	190491	1.64%	1	1	138	8019	1.72%
AML 132	Plasma	0 month	Minus 7	130	37	35	94.6%	22523	295005	7.63%	1	0	0	9845	0.00%
AML 132	Plasma	2 months	56	130	37	28	75.7%	96	257548	0.04%	1	0	0	9732	0.00%
AML 132	Plasma	3 months	81	130	37	35	94.6%	179	197259	0.09%	1	0	0	7144	0.00%
AML 132	Plasma	6 months	172	130	37	36	97.3%	49051	245092	20.01%	1	0	0	8343	0.00%
AML 133	Peripheral blood	Diagnostic	Minus 118	71	52	52	100.0%	96023	210867	45.54%	4	4	11414	21791	52.38%
AML 133	Peripheral blood	Remission	Minus 71	71	52	44	84.6%	512	461181	0.11%	4	4	46	52154	0.09%
AML 133	Plasma	2 months	55	71	52	49	94.2%	547	851764	0.06%	4	4	55	73162	0.08%
AML 133	Plasma	6 months	190	71	52	51	98.1%	173381	748863	23.15%	4	4	16781	61899	27.11%
AML 134	Bone Marrow	Diagnostic	Minus 336	60	39	39	100.0%	24540	69658	35.23%	1	1	1516	2122	71.44%
AML 134	Bone Marrow	Remission	Minus 205	60	39	38	97.4%	2474	228936	1.08%	1	1	122	6396	1.91%
AML 134	Plasma	0 month	Minus 7	60	39	39	100.0%	84066	230508	36.47%	1	1	4739	6747	70.24%
AML 134	Plasma	2 months	58	60	39	38	97.4%	378	296811	0.13%	1	1	25	9412	0.27%
AML 134	Plasma	3 months	85	60	39	38	97.4%	5041	297184	1.70%	1	1	251	8823	2.84%
AML 134	Plasma	6 months	189	60	39	3	7.7%	3	194220	0.00%	1	0	0	5636	0.00%
AML 134	Plasma	12 months	363	60	39	4	10.3%	4	555521	0.00%	1	1	1	18022	0.01%
AML 235	Bone marrow	Diagnostic	Minus 130	60	82	82	100.0%	135559	359181	37.74%	3	3	1397	9146	15.27%
AML 235	Bone marrow	Remission	Minus 94	60	82	35	42.7%	192	594207	0.03%	3	0	0	15894	0.00%
AML 235	Plasma	2 months	55	60	82	75	91.5%	384	3573548	0.01%	3	3	7	124829	0.01%
AML 235	Plasma	3 months	84	60	82	61	74.4%	161	1562501	0.01%	3	1	2	54452	0.00%
AML 235	Plasma	6 months	196	60	82	66	80.5%	208	837424	0.02%	3	1	2	28231	0.01%
AML 235	Plasma	12 months	368	60	82	8	9.8%	15	319604	0.00%	3	1	1	11342	0.01%
AML 236	Bone marrow	Diagnostic	Minus 152	60	12	12	100.0%	1270	48807	2.60%	1	1	136	3269	4.16%
AML 236	Bone marrow	Remission	Minus 57	60	12	12	100.0%	1833	135814	1.35%	1	1	1	6581	0.02%
AML 236	Plasma	2 months	57	60	12	11	91.7%	83	669929	0.01%	1	1	17	38417	0.04%
AML 236	Plasma	6 months	186	60	12	5	41.7%	3299	244871	1.35%	1	1	637	14416	4.42%
AML 237	Bone marrow	Diagnostic	Minus 206	180	82	82	100.0%	353072	779580	45.29%	2	2	4737	9884	47.93%
AML 237	Bone marrow	Remission	Minus 61	180	82	43	52.4%	944	591339	0.16%	2	1	23	7531	0.31%
AML 237	Plasma	2 months	60	180	82	11	13.4%	13	4948179	0.00%	2	1	1	50514	0.00%
AML 237	Plasma	6 months	175	180	82	8	9.8%	9	2470781	0.00%	2	1	1	29587	0.00%
AML 238	Bone marrow	Diagnostic	Minus 140	138	81	81	100.0%	130741	365619	35.76%	1	1	1418	3978	35.65%
AML 238	Bone marrow	Remission	Minus 92	138	81	69	85.2%	1193	561561	0.21%	1	1	6	6043	0.10%
AML 238	Plasma	2 months	62	138	81	80	98.8%	4532	3998171	0.11%	1	1	31	38724	0.08%
AML 238	Plasma	6 months	163	138	81	47	58.0%	83	668128	0.01%	1	1	1	6664	0.02%
AML 238	Plasma	12 months	404	138	81	1	1.2%	1	620635	0.00%	1	0	0	6096	0.00%
AML 239	Bone marrow	Diagnostic	Minus 173	90	82	82	100.0%	150649	365340	41.24%	3	3	3100	7749	40.01%
AML 239	Bone marrow	Remission	Minus 37	90	82	26	31.7%	485	927231	0.05%	3	1	882	18589	4.74%
AML 239	Plasma	0 month	Minus 7	90	82	18	22.0%	95	119807	0.08%	3	1	139	3208	4.33%
AML 239	Plasma	2 months	57	90	82	2	2.4%	2	2026974	0.00%	3	1	6	42486	0.01%
AML 239	Plasma	6 months	181	90	82	0	0.0%	0	194568	0.00%	3	0	0	3068	0.00%
AML 239	Plasma	3 months	83	90	82	2	2.4%	2	334186	0.00%	3	1	2	7982	0.03%
AML 239	Plasma	12 months	349	90	82	3	3.7%	4	1646012	0.00%	3	1	1	27187	0.00%
AML 240	Bone marrow	Diagnostic	Minus 177	57	67	67	100.0%	271154	636620	42.59%	2	2	2859	7803	36.64%
AML 240	Bone marrow	Remission	Minus 33	57	67	14	20.9%	20	331094	0.01%	2	0	0	4701	0.00%
AML 240	Plasma	0 month	Minus 7	57	67	60	89.6%	264	1632661	0.02%	2	0	0	19580	0.00%

AML 240	Plasma	2 months	56	57	67	51	76.1%	129	2492893	0.01%	2	0	0	30332	0.00%
AML 240	Plasma	3 months	82	57	67	47	70.1%	121	3009828	0.00%	2	0	0	18209	0.00%
AML 240	Plasma	6 months	173	57	67	19	28.4%	23	1494422	0.00%	2	0	0	21475	0.00%
AML 240	Plasma	12 months	363	57	67	0	0.0%	0	713527	0.00%	2	0	0	10154	0.00%
AML 241	Bone marrow	Diagnostic	Minus 475	60	80	80	100.0%	171249	407242	42.05%	1	1	782	1715	45.60%
AML 241	Bone marrow	Remission	Minus 29	60	80	46	57.5%	92	165661	0.06%	1	1	1	636	0.16%
AML 241	Plasma	0 month	Minus 7	60	80	18	22.5%	20	525778	0.00%	1	0	0	3767	0.00%
AML 241	Plasma	2 months	57	60	80	3	3.8%	3	2869033	0.00%	1	0	0	7441	0.00%
AML 241	Plasma	3 months	92	60	80	2	2.5%	2	1482407	0.00%	1	0	0	9483	0.00%
AML 241	Plasma	6 months	177	60	80	2	2.5%	2	2207813	0.00%	1	0	0	11873	0.00%
AML 241	Plasma	12 months	358	60	80	0	0.0%	0	842984	0.00%	1	0	0	5304	0.00%
AML 242	Bone marrow	Diagnostic	Minus 507	106	69	69	100.0%	211538	591138	35.78%	7	7	7613	34296	22.20%
AML 242	Bone marrow	Remission	Minus 44	106	69	37	53.6%	1275	299889	0.43%	7	1	938	20656	4.54%
AML 242	Plasma	2 months	56	106	69	28	40.6%	240	4658767	0.01%	7	1	85	187692	0.05%
AML 242	Plasma	3 months	106	106	69	18	26.1%	33	862469	0.00%	7	1	9	48198	0.02%
AML 242	Plasma	6 months	180	106	69	37	53.6%	1720	669814	0.26%	7	1	12	11564	0.10%
AML 242	Plasma	12 months	376	106	69	1	1.4%	1	1640073	0.00%	7	0	0	88203	0.00%
AML 244	Bone marrow	Diagnostic	Minus 149	60	83	83	100.0%	323547	1335278	24.23%	3	3	2737	25698	10.65%
AML 244	Bone marrow	Remission	Minus 43	60	83	65	78.3%	161	1145059	0.01%	3	0	0	17160	0.00%
AML 244	Plasma	2 months	66	60	83	81	97.6%	898	3040498	0.03%	3	2	8	81306	0.01%
AML 244	Plasma	6 months	192	60	83	2	2.4%	2	1585052	0.00%	3	0	0	44920	0.00%
AML 244	Plasma	12 months	360	60	83	1	1.2%	1	858010	0.00%	3	0	0	25457	0.00%
AML 245	Bone marrow	Diagnostic	Minus 137	57	71	71	100.0%	126917	299142	42.43%	4	4	4544	11475	39.60%
AML 245	Bone marrow	Remission	Minus 106	57	71	58	81.7%	138	411654	0.03%	4	1	1	14206	0.01%
AML 245	Plasma	0 month	Minus 7	57	71	66	93.0%	276	117576	0.23%	4	4	40	6001	0.67%
AML 245	Plasma	2 months	63	57	71	64	90.1%	243	80238	0.30%	4	4	23	4081	0.56%
AML 245	Plasma	3 months	92	57	71	4	5.6%	4	305794	0.00%	4	1	38	11446	0.33%
AML 246	Bone marrow	Diagnostic	Minus 150	60	74	74	100.0%	31694	498167	6.36%	1	1	216	1997	10.82%
AML 246	Bone marrow	Remission	Minus 65	60	74	46	62.2%	87	443131	0.02%	1	1	1	1079	0.09%
AML 246	Plasma	0 month	Minus 7	60	74	15	20.3%	16	361933	0.00%	1	0	0	1901	0.00%
AML 246	Plasma	2 months	55	60	74	41	55.4%	57	4596750	0.00%	1	0	0	15962	0.00%
AML 246	Plasma	6 months	176	60	74	54	73.0%	128	2274087	0.01%	1	0	0	9745	0.00%
AML 246	Plasma	12 months	358	60	74	19	25.7%	21	2181994	0.00%	1	0	0	8410	0.00%
AML 247	Bone marrow	Diagnostic	Minus 297	60	79	79	100.0%	447998	1131065	39.61%	6	6	3144	54325	5.79%
AML 247	Bone marrow	Remission	Minus 161	60	79	9	11.4%	11	658314	0.00%	6	0	0	31284	0.00%
AML 247	Plasma	2 months	57	60	79	55	69.6%	137	171274	0.08%	6	0	0	10549	0.00%
AML 247	Plasma	3 months	90	60	79	71	89.9%	9747	325407	3.00%	6	1	89	20100	0.44%
AML 248	Bone marrow	Diagnostic	Minus 106	60	81	81	100.0%	290830	722169	40.27%	1	1	18	7317	0.25%
AML 248	Bone marrow	Remission	Minus 16	60	81	73	90.1%	3053	779472	0.39%	1	1	418	8095	5.16%
AML 248	Plasma	0 month	Minus 7	60	81	71	87.7%	9768	1786852	0.55%	1	1	1169	19534	5.98%
AML 248	Plasma	2 months	58	60	81	69	85.2%	4211	4496633	0.09%	1	1	27	47151	0.06%
AML 248	Plasma	3 months	86	60	81	60	74.1%	1232	1132386	0.11%	1	0	0	11932	0.00%
AML 249	Bone marrow	Diagnostic	Minus 194	66	32	32	100.0%	8129	25295	32.14%	4	4	1337	6839	19.55%
AML 249	Bone marrow	Remission	Minus 40	66	32	28	87.5%	369	319360	0.12%	4	3	35	26977	0.13%
AML 249	Plasma	2 months	56	66	32	24	75.0%	232	1283117	0.02%	4	1	7	90536	0.01%
AML 249	Plasma	6 months	175	66	32	3	9.4%	3	1555988	0.00%	4	1	1	94860	0.00%
AML 249	Plasma	12 months	353	66	32	0	0.0%	0	503479	0.00%	4	0	0	38113	0.00%
AML 250	Bone marrow	Diagnostic	Minus 126	70	81	81	100.0%	281438	723737	38.89%	3	3	4022	12116	33.20%
AML 250	Bone marrow	Remission	Minus 92	70	81	35	43.2%	93	745551	0.01%	3	1	3	11831	0.03%
AML 250	Plasma	0 month	Minus 7	70	81	40	49.4%	103	748942	0.01%	3	1	1	12550	0.01%
AML 250	Plasma	2 months	56	70	81	4	4.9%	4	635648	0.00%	3	0	0	10785	0.00%
AML 250	Plasma	3 months	87	70	81	5	6.2%	7	1150663	0.00%	3	0	0	18241	0.00%
AML 250	Plasma	6 months	172	70	81	5	6.2%	6	1097866	0.00%	3	0	0	17907	0.00%
AML 250	Plasma	12 months	413	70	81	0	0.0%	0	1714050	0.00%	3	0	0	28879	0.00%
AML 251	Bone marrow	Diagnostic	Minus 147	180	76	76	100.0%	83473	351865	23.72%	3	3	3207	13603	23.58%
AML 251	Bone marrow	Remission	Minus 38	180	76	32	42.1%	60	566576	0.01%	3	1	5	15001	0.03%
AML 251	Plasma	0 month	Minus 7	180	76	75	98.7%	1228	2643761	0.05%	3	3	50	84460	0.06%
AML 251	Plasma	2 months	56	180	76	36	47.4%	53	4523621	0.00%	3	1	2	139909	0.00%
AML 251	Plasma	3 months	87	180	76	45	59.2%	91	2822566	0.00%	3	1	4	82156	0.00%
AML 251	Plasma	6 months	176	180	76	5	6.6%	7	4660989	0.00%	3	0	0	152458	0.00%
AML 251	Plasma	12 months	351	180	76	1	1.3%	1	1028218	0.00%	3	0	0	37243	0.00%

NA = data not available; PB = peripheral blood; CD3 = CD3 sorted T cells

Table S3: Mutations identified in paired plasma and bone marrow samples at remission and ~3 months following transplantation.

Patient	Mutation	Timepoint	Days post-alloHCT, plasma collection	Number of mutant molecules in plasma	Total number of molecules assessed in plasma	Mutant allele frequency in plasma	Days post-alloHCT, bone marrow collection	Number of mutant molecules in bone marrow	Total number of molecules assessed in bone marrow	Mutant allele frequency in bone marrow
AML 121	chr1:110245902G>A	Remission	Minus 7	0	5204	0.000%	Minus 182	127	6997	1.815%
AML 121	chr10:19159747A>G	Remission	Minus 7	0	407	0.000%	Minus 182	17	1096	1.551%
AML 121	chr10:63867282G>A	Remission	Minus 7	68	5125	1.327%	Minus 182	61	6498	0.939%
AML 121	chr10:75654454G>A	Remission	Minus 7	1	948	0.105%	Minus 182	26	2005	1.297%
AML 121	chr11:21137971G>A	Remission	Minus 7	5	4760	0.105%	Minus 182	129	7643	1.688%
AML 121	chr11:28565424C>T	Remission	Minus 7	17	4246	0.400%	Minus 182	12	5656	0.212%
AML 121	chr11:41711884C>T	Remission	Minus 7	4	4080	0.098%	Minus 182	98	6409	1.529%
AML 121	chr11:45984682C>T	Remission	Minus 7	65	5048	1.288%	Minus 182	71	7039	1.009%
AML 121	chr12:101081568A>G	Remission	Minus 7	1	4092	0.024%	Minus 182	134	6147	2.180%
AML 121	chr13:113254106G>A	Remission	Minus 7	72	5643	1.276%	Minus 182	71	7491	0.948%
AML 121	chr13:35209302C>T	Remission	Minus 7	2	3471	0.058%	Minus 182	92	4232	2.174%
AML 121	chr14:77501038C>T	Remission	Minus 7	64	4696	1.363%	Minus 182	86	7123	1.207%
AML 121	chr15:41955386G>A	Remission	Minus 7	3	5751	0.052%	Minus 182	111	7682	1.445%
AML 121	chr15:42068543C>T	Remission	Minus 7	1	6588	0.015%	Minus 182	112	7333	1.527%
AML 121	chr15:59271645T>C	Remission	Minus 7	2	3236	0.062%	Minus 182	52	4896	1.062%
AML 121	chr16:61878846T>G	Remission	Minus 7	1	4312	0.023%	Minus 182	89	6904	1.289%
AML 121	chr16:79606865T>C	Remission	Minus 7	48	3762	1.276%	Minus 182	47	5303	0.886%
AML 121	chr17:12323012T>C	Remission	Minus 7	2	4410	0.045%	Minus 182	116	7428	1.562%
AML 121	chr18:33220934C>T	Remission	Minus 7	1	5154	0.019%	Minus 182	114	6997	1.629%
AML 121	chr18:51651640C>G	Remission	Minus 7	71	5045	1.407%	Minus 182	67	6904	0.970%
AML 121	chr2:104729661C>G	Remission	Minus 7	666	6770	9.838%	Minus 182	477	6757	7.059%
AML 121	chr2:71464109C>T	Remission	Minus 7	42	3768	1.115%	Minus 182	58	5819	0.997%
AML 121	chr20:57195801C>T	Remission	Minus 7	104	6100	1.705%	Minus 182	94	8271	1.137%
AML 121	chr20:62775175G>A	Remission	Minus 7	104	7068	1.471%	Minus 182	96	9027	1.063%
AML 121	chr3:115810896G>A	Remission	Minus 7	3	4860	0.062%	Minus 182	117	7111	1.645%
AML 121	chr3:176246793T>C	Remission	Minus 7	2	3722	0.054%	Minus 182	113	5945	1.901%
AML 121	chr3:94302682C>T	Remission	Minus 7	0	4229	0.000%	Minus 182	102	5765	1.769%
AML 121	chr4:134547370G>C	Remission	Minus 7	3	4592	0.065%	Minus 182	53	6600	0.803%
AML 121	chr4:147093498G>A	Remission	Minus 7	2	2943	0.068%	Minus 182	33	3370	0.979%
AML 121	chr4:154043601G>C	Remission	Minus 7	1	5603	0.018%	Minus 182	61	6597	0.925%
AML 121	chr5:15219770G>A	Remission	Minus 7	0	5108	0.000%	Minus 182	131	6703	1.954%
AML 121	chr5:2216594G>A	Remission	Minus 7	50	3719	1.344%	Minus 182	50	4528	1.104%
AML 121	chr5:8331558G>A	Remission	Minus 7	8	637	1.256%	Minus 182	12	1492	0.804%
AML 121	chr5:89300749C>G	Remission	Minus 7	1	3874	0.026%	Minus 182	100	5559	1.799%
AML 121	chr7:11262755A>C	Remission	Minus 7	3	4981	0.060%	Minus 182	42	6015	0.698%
AML 121	chr7:130141280T>C	Remission	Minus 7	4	1539	0.260%	Minus 182	3	2907	0.103%
AML 121	chr7:154705495C>T	Remission	Minus 7	18	5553	0.324%	Minus 182	25	6864	0.364%
AML 121	chr7:35356833C>T	Remission	Minus 7	2	3430	0.058%	Minus 182	35	5360	0.653%
AML 121	chr8:102896093A>G	Remission	Minus 7	3	3908	0.077%	Minus 182	42	5999	0.700%
AML 121	chr8:127644825G>A	Remission	Minus 7	85	6923	1.228%	Minus 182	83	8411	0.987%
AML 121	chr8:22624337T>C	Remission	Minus 7	16	1017	1.573%	Minus 182	22	2166	1.016%
AML 121	chr9:135723685C>T	Remission	Minus 7	26	5384	0.483%	Minus 182	16	7183	0.223%
AML 121	chr9:68940919G>C	Remission	Minus 7	85	4870	1.745%	Minus 182	72	6527	1.103%
AML 121	chrX:50994220G>A	Remission	Minus 7	6	3027	0.198%	Minus 182	128	3923	3.263%

AML 121	chrX:64270054C>T	Remission	Minus 7	2	1547	0.129%	Minus 182	37	3058	1.210%
AML 124	chr1:193497400C>G	Remission	Minus 7	1	2419	0.041%	Minus 126	0	5683	0.000%
AML 124	chr3:191874226T>C	Remission	Minus 7	0	1899	0.000%	Minus 126	1	4961	0.020%
AML 126	chr1:219518740C>G	Remission	Minus 7	0	6962	0.000%	Minus 107	5	7364	0.068%
AML 126	chr10:1167403T>C	Remission	Minus 7	5	9605	0.052%	Minus 107	1	6756	0.015%
AML 126	chr16:79739452A>G	Remission	Minus 7	0	966	0.000%	Minus 107	1	1975	0.051%
AML 126	chr19:46015947T>C	Remission	Minus 7	0	8405	0.000%	Minus 107	1	8394	0.012%
AML 126	chr2:160373535C>G	Remission	Minus 7	4	4756	0.084%	Minus 107	0	7446	0.000%
AML 126	chr2:25234373C>T	Remission	Minus 7	4	10797	0.037%	Minus 107	3	8577	0.035%
AML 126	chr2:60499249C>G	Remission	Minus 7	8	12400	0.065%	Minus 107	5	8674	0.058%
AML 126	chr5:139697924T>G	Remission	Minus 7	1	11249	0.009%	Minus 107	2	7117	0.028%
AML 126	chr5:156738149A>G	Remission	Minus 7	1	2995	0.033%	Minus 107	0	4196	0.000%
AML 126	chr6:82647125A>G	Remission	Minus 7	1	1100	0.091%	Minus 107	1	2031	0.049%
AML 126	chr7:154503700G>-	Remission	Minus 7	3	8651	0.035%	Minus 107	2	6994	0.029%
AML 126	chr7:68016075C>G	Remission	Minus 7	6	9231	0.065%	Minus 107	4	7943	0.050%
AML 129	chr10:124991078G>C	Remission	Minus 7	1	7061	0.014%	Minus 99	0	6849	0.000%
AML 129	chr11:133840616A>G	Remission	Minus 7	1	6893	0.015%	Minus 99	0	5966	0.000%
AML 129	chr19:10877193A>G	Remission	Minus 7	1	9546	0.010%	Minus 99	0	9034	0.000%
AML 129	chr21:34880577A>C	Remission	Minus 7	0	8160	0.000%	Minus 99	1	9757	0.010%
AML 129	chr4:105272753G>T	Remission	Minus 7	2	8667	0.023%	Minus 99	0	10015	0.000%
AML 129	chr4:105272754C>A	Remission	Minus 7	2	8586	0.023%	Minus 99	0	9943	0.000%
AML 129	chr5:173670181T>G	Remission	Minus 7	0	7083	0.000%	Minus 99	1	10722	0.009%
AML 129	chr6:104047120C>G	Remission	Minus 7	1	7413	0.013%	Minus 99	1	10150	0.010%
AML 129	chr6:86337232A>C	Remission	Minus 7	1	7935	0.013%	Minus 99	1	10019	0.010%
AML 129	chr9:129875614A>G	Remission	Minus 7	1	9279	0.011%	Minus 99	0	7287	0.000%
AML 130	chr1:106285578T>G	Remission	Minus 7	3	6302	0.048%	Minus 127	0	9672	0.000%
AML 130	chr1:166135924A>G	Remission	Minus 7	4	7216	0.055%	Minus 127	1	8201	0.012%
AML 130	chr10:125125474T>C	Remission	Minus 7	2	6219	0.032%	Minus 127	0	7788	0.000%
AML 130	chr10:34621587A>T	Remission	Minus 7	334	6990	4.778%	Minus 127	514	12517	4.106%
AML 130	chr10:85852159T>C	Remission	Minus 7	6	8882	0.068%	Minus 127	1	10500	0.010%
AML 130	chr11:122692233A>G	Remission	Minus 7	325	6301	5.158%	Minus 127	368	8267	4.451%
AML 130	chr11:38802150A>G	Remission	Minus 7	3	6678	0.045%	Minus 127	0	4780	0.000%
AML 130	chr11:64395060A>G	Remission	Minus 7	2	5929	0.034%	Minus 127	2	8617	0.023%
AML 130	chr12:104741654A>G	Remission	Minus 7	7	7523	0.093%	Minus 127	11	7364	0.149%
AML 130	chr12:13896169A>G	Remission	Minus 7	3	7374	0.041%	Minus 127	0	9672	0.000%
AML 130	chr12:92614953T>G	Remission	Minus 7	12	7668	0.156%	Minus 127	14	10509	0.133%
AML 130	chr13:55240615T>C	Remission	Minus 7	2	6405	0.031%	Minus 127	0	8319	0.000%
AML 130	chr14:26683707T>C	Remission	Minus 7	356	6216	5.727%	Minus 127	385	8789	4.380%
AML 130	chr14:48973412A>G	Remission	Minus 7	1	6629	0.015%	Minus 127	0	9297	0.000%
AML 130	chr14:90423441C>T	Remission	Minus 7	1193	7498	15.911%	Minus 127	2251	15277	14.735%
AML 130	chr14:98255192T>C	Remission	Minus 7	1350	8521	15.843%	Minus 127	1952	13276	14.703%
AML 130	chr16:46743605A>G	Remission	Minus 7	163	3242	5.028%	Minus 127	152	3698	4.110%
AML 130	chr16:65528696G>C	Remission	Minus 7	563	6785	8.298%	Minus 127	990	12806	7.731%
AML 130	chr17:59314673A>G	Remission	Minus 7	4	6075	0.066%	Minus 127	0	6309	0.000%
AML 130	chr18:51567834A>T	Remission	Minus 7	42	6420	0.654%	Minus 127	55	9928	0.554%
AML 130	chr2:119166886A>C	Remission	Minus 7	3	5311	0.056%	Minus 127	1	11752	0.009%
AML 130	chr2:80091791A>G	Remission	Minus 7	4	6877	0.058%	Minus 127	2	9251	0.022%
AML 130	chr20:11727205C>G	Remission	Minus 7	24	6066	0.396%	Minus 127	32	10081	0.317%
AML 130	chr3:128483911->TA	Remission	Minus 7	1	7039	0.014%	Minus 127	0	14528	0.000%
AML 130	chr3:66126348G>C	Remission	Minus 7	6	7792	0.077%	Minus 127	1	12776	0.008%

AML 130	chr4:64227897T>A	Remission	Minus 7	0	6023	0.000%	Minus 127	2	9441	0.021%
AML 130	chr4:94032696A>G	Remission	Minus 7	3	7286	0.041%	Minus 127	0	8280	0.000%
AML 130	chr5:178289367T>C	Remission	Minus 7	5	9386	0.053%	Minus 127	1	5948	0.017%
AML 130	chr5:56376041C>G	Remission	Minus 7	25	8134	0.307%	Minus 127	14	9666	0.145%
AML 130	chr6:2469835T>C	Remission	Minus 7	5	8899	0.056%	Minus 127	3	12716	0.024%
AML 130	chr6:722843T>C	Remission	Minus 7	3	10034	0.030%	Minus 127	2	9509	0.021%
AML 130	chr6:84468378G>C	Remission	Minus 7	1	6187	0.016%	Minus 127	0	10085	0.000%
AML 130	chr7:11319387A>G	Remission	Minus 7	1	7663	0.013%	Minus 127	0	10053	0.000%
AML 130	chr7:135976147G>C	Remission	Minus 7	2	6097	0.033%	Minus 127	1	9134	0.011%
AML 130	chr9:78680128G>A	Remission	Minus 7	4	8018	0.050%	Minus 127	0	9861	0.000%
AML 132	chr1:117114058A>G	Remission	Minus 7	510	6968	7.319%	Minus 103	47	2634	1.784%
AML 132	chr1:14930392A>G	Remission	Minus 7	473	7448	6.351%	Minus 103	31	3132	0.990%
AML 132	chr1:188189173A>G	Remission	Minus 7	500	7272	6.876%	Minus 103	73	4589	1.591%
AML 132	chr1:53650861A>G	Remission	Minus 7	613	8661	7.078%	Minus 103	73	5438	1.342%
AML 132	chr1:86765225T>C	Remission	Minus 7	609	8444	7.212%	Minus 103	85	6104	1.393%
AML 132	chr10:127095321C>G	Remission	Minus 7	879	11512	7.636%	Minus 103	105	7210	1.456%
AML 132	chr11:101322094T>C	Remission	Minus 7	1016	7446	13.645%	Minus 103	122	4557	2.677%
AML 132	chr11:96259212C>G	Remission	Minus 7	3386	10724	31.574%	Minus 103	445	5700	7.807%
AML 132	chr14:34361569A>G	Remission	Minus 7	553	7856	7.039%	Minus 103	67	5505	1.217%
AML 132	chr14:82859772T>A	Remission	Minus 7	521	8551	6.093%	Minus 103	59	4952	1.191%
AML 132	chr15:69978580A>G	Remission	Minus 7	700	9580	7.307%	Minus 103	96	6558	1.464%
AML 132	chr16:54566631C>G	Remission	Minus 7	660	9126	7.232%	Minus 103	87	7168	1.214%
AML 132	chr17:75109062A>G	Remission	Minus 7	771	9779	7.884%	Minus 103	176	6503	2.706%
AML 132	chr17:7676011T>C	Remission	Minus 7	0	9845	0.000%	Minus 103	138	8019	1.721%
AML 132	chr21:36858963T>G	Remission	Minus 7	630	9362	6.729%	Minus 103	101	7090	1.425%
AML 132	chr3:150683017C>G	Remission	Minus 7	733	10241	7.158%	Minus 103	67	6009	1.115%
AML 132	chr4:18228019T>A	Remission	Minus 7	361	4597	7.853%	Minus 103	41	2822	1.453%
AML 132	chr4:187358097T>A	Remission	Minus 7	524	6951	7.538%	Minus 103	82	5621	1.459%
AML 132	chr5:180685022C>G	Remission	Minus 7	639	8407	7.601%	Minus 103	83	6888	1.205%
AML 132	chr5:30956787C>G	Remission	Minus 7	197	3676	5.359%	Minus 103	27	2202	1.226%
AML 132	chr5:79668936T>C	Remission	Minus 7	478	6076	7.867%	Minus 103	54	3925	1.376%
AML 132	chr5:95733819A>G	Remission	Minus 7	626	8288	7.553%	Minus 103	68	5372	1.266%
AML 132	chr6:129060397A>G	Remission	Minus 7	488	7449	6.551%	Minus 103	51	3482	1.465%
AML 132	chr6:14230036G>C	Remission	Minus 7	854	11514	7.417%	Minus 103	109	7728	1.410%
AML 132	chr6:41418913G>C	Remission	Minus 7	726	10169	7.139%	Minus 103	84	5462	1.538%
AML 132	chr7:39181631A>G	Remission	Minus 7	597	9101	6.560%	Minus 103	102	6667	1.530%
AML 132	chr8:11125590A>G	Remission	Minus 7	179	9661	1.853%	Minus 103	33	5962	0.554%
AML 132	chr8:35002504T>C	Remission	Minus 7	112	1874	5.977%	Minus 103	59	4177	1.412%
AML 132	chr8:35006381A>G	Remission	Minus 7	398	5830	6.827%	Minus 103	61	4668	1.307%
AML 132	chr8:95269782A>G	Remission	Minus 7	277	6340	4.369%	Minus 103	56	4460	1.256%
AML 132	chr9:127135456A>G	Remission	Minus 7	605	8798	6.877%	Minus 103	58	3262	1.778%
AML 132	chr9:79508916A>G	Remission	Minus 7	634	8992	7.051%	Minus 103	68	4966	1.369%
AML 132	chr9:86678564C>G	Remission	Minus 7	568	8649	6.567%	Minus 103	81	5923	1.368%
AML 132	chrX:28806706A>G	Remission	Minus 7	686	9690	7.079%	Minus 103	78	5400	1.444%
AML 132	chrX:39806574T>A	Remission	Minus 7	402	6747	5.958%	Minus 103	61	4580	1.332%
AML 132	chrX:44912774T>C	Remission	Minus 7	618	9381	6.588%	Minus 103	90	5711	1.576%
AML 134	chr1:34198846A>G	Remission	Minus 7	2517	7093	35.486%	Minus 205	75	6530	1.149%
AML 134	chr1:43339489T>C	Remission	Minus 7	4739	6747	70.239%	Minus 205	122	6396	1.907%
AML 134	chr10:132641668T>A	Remission	Minus 7	2034	5714	35.597%	Minus 205	62	6223	0.996%
AML 134	chr10:64087228A>G	Remission	Minus 7	2733	7456	36.655%	Minus 205	65	6526	0.996%

AML 134	chr11:126659511C>G	Remission	Minus 7	1826	5913	30.881%	Minus 205	70	6595	1.061%
AML 134	chr11:30454130T>C	Remission	Minus 7	2111	6500	32.477%	Minus 205	57	5857	0.973%
AML 134	chr11:84494225A>C	Remission	Minus 7	2314	6066	38.147%	Minus 205	65	6146	1.058%
AML 134	chr12:12228999G>C	Remission	Minus 7	2387	6626	36.025%	Minus 205	66	6681	0.988%
AML 134	chr12:17646823A>G	Remission	Minus 7	2525	6959	36.284%	Minus 205	85	7114	1.195%
AML 134	chr12:30901554T>C	Remission	Minus 7	2299	6528	35.218%	Minus 205	57	5300	1.075%
AML 134	chr13:20025322A>G	Remission	Minus 7	2338	6458	36.203%	Minus 205	62	5686	1.090%
AML 134	chr13:41307073T>G	Remission	Minus 7	2155	5893	36.569%	Minus 205	71	7016	1.012%
AML 134	chr14:31515609T>A	Remission	Minus 7	1876	5119	36.648%	Minus 205	51	6482	0.787%
AML 134	chr14:82732502T>A	Remission	Minus 7	1883	5202	36.198%	Minus 205	67	6346	1.056%
AML 134	chr15:95819504C>G	Remission	Minus 7	1906	5325	35.793%	Minus 205	68	5998	1.134%
AML 134	chr16:27829998T>C	Remission	Minus 7	2474	6923	35.736%	Minus 205	63	6117	1.030%
AML 134	chr16:49111324A>T	Remission	Minus 7	2701	7524	35.898%	Minus 205	73	7466	0.978%
AML 134	chr17:62783058A>G	Remission	Minus 7	3034	7084	42.829%	Minus 205	91	6253	1.455%
AML 134	chr2:110840630C>A	Remission	Minus 7	2634	7302	36.072%	Minus 205	80	7019	1.140%
AML 134	chr2:136910168A>G	Remission	Minus 7	2196	5951	36.901%	Minus 205	48	5392	0.890%
AML 134	chr2:150341865T>A	Remission	Minus 7	1608	4457	36.078%	Minus 205	51	5606	0.910%
AML 134	chr2:177636241A>G	Remission	Minus 7	1942	5476	35.464%	Minus 205	65	6141	1.058%
AML 134	chr2:191150948T>C	Remission	Minus 7	2119	6152	34.444%	Minus 205	60	5927	1.012%
AML 134	chr2:192779344T>C	Remission	Minus 7	1640	4247	38.615%	Minus 205	49	4142	1.183%
AML 134	chr2:206738778A>G	Remission	Minus 7	480	6493	7.393%	Minus 205	18	6317	0.285%
AML 134	chr20:16765363A>G	Remission	Minus 7	1895	5309	35.694%	Minus 205	59	5283	1.117%
AML 134	chr20:55996573A>T	Remission	Minus 7	1920	5176	37.094%	Minus 205	71	6151	1.154%
AML 134	chr3:111893509A>G	Remission	Minus 7	8	11	72.727%	Minus 205	0	12	0.000%
AML 134	chr3:146163035A>G	Remission	Minus 7	1781	5070	35.128%	Minus 205	67	5234	1.280%
AML 134	chr4:163363495T>C	Remission	Minus 7	1539	4324	35.592%	Minus 205	60	4767	1.259%
AML 134	chr4:180595890T>C	Remission	Minus 7	2602	6729	38.668%	Minus 205	60	5809	1.033%
AML 134	chr4:2804783G>C	Remission	Minus 7	2832	7844	36.104%	Minus 205	77	6717	1.146%
AML 134	chr5:15522182C>G	Remission	Minus 7	1011	4603	21.964%	Minus 205	34	5451	0.624%
AML 134	chr6:107563734T>C	Remission	Minus 7	1619	4326	37.425%	Minus 205	71	4906	1.447%
AML 134	chr7:28768132T>A	Remission	Minus 7	3928	8289	47.388%	Minus 205	72	6457	1.115%
AML 134	chr9:27906493A>G	Remission	Minus 7	1877	5018	37.405%	Minus 205	56	5125	1.093%
AML 134	chr9:70386318T>A	Remission	Minus 7	2020	5765	35.039%	Minus 205	66	5880	1.122%
AML 134	chr9:81676483C>G	Remission	Minus 7	2057	5859	35.108%	Minus 205	68	5723	1.188%
AML 134	chr9:92960287T>C	Remission	Minus 7	2506	6977	35.918%	Minus 205	72	6145	1.172%
AML 239	chr1:110007539G>C	Remission	Minus 7	7	1507	0.464%	Minus 37	26	14604	0.178%
AML 239	chr1:221759682T>C	Remission	Minus 7	1	1276	0.078%	Minus 37	7	10892	0.064%
AML 239	chr1:237763374C>T	Remission	Minus 7	0	1612	0.000%	Minus 37	6	13152	0.046%
AML 239	chr11:112858841G>A	Remission	Minus 7	0	1703	0.000%	Minus 37	5	12150	0.041%
AML 239	chr12:115131646C>T	Remission	Minus 7	0	1278	0.000%	Minus 37	5	11740	0.043%
AML 239	chr12:63013504G>A	Remission	Minus 7	8	1369	0.584%	Minus 37	21	11043	0.190%
AML 239	chr12:78870588G>A	Remission	Minus 7	2	1274	0.157%	Minus 37	1	9825	0.010%
AML 239	chr12:96679905G>C	Remission	Minus 7	3	1427	0.210%	Minus 37	4	10762	0.037%
AML 239	chr13:99956664->T	Remission	Minus 7	1	1706	0.059%	Minus 37	6	15723	0.038%
AML 239	chr16:52026432C>T	Remission	Minus 7	0	1186	0.000%	Minus 37	1	4710	0.021%
AML 239	chr18:3655216G>A	Remission	Minus 7	0	1449	0.000%	Minus 37	1	13022	0.008%
AML 239	chr2:232975430C>T	Remission	Minus 7	1	1261	0.079%	Minus 37	1	1036	0.097%
AML 239	chr3:184236681C>T	Remission	Minus 7	4	1340	0.299%	Minus 37	23	11405	0.202%
AML 239	chr3:27294537G>A	Remission	Minus 7	1	1350	0.074%	Minus 37	1	15432	0.006%
AML 239	chr4:159389505A>T	Remission	Minus 7	7	1553	0.451%	Minus 37	34	15583	0.218%

AML 239	chr4:65559515A>G	Remission	Minus 7	1	1131	0.088%	Minus 37	5	7787	0.064%
AML 239	chr4:71493502T>C	Remission	Minus 7	4	878	0.456%	Minus 37	17	12034	0.141%
AML 239	chr5:153490230T>C	Remission	Minus 7	3	1392	0.216%	Minus 37	13	13141	0.099%
AML 239	chr6:133191380C>T	Remission	Minus 7	0	1432	0.000%	Minus 37	1	11002	0.009%
AML 239	chr6:70101478G>C	Remission	Minus 7	40	1056	3.788%	Minus 37	227	7227	3.141%
AML 239	chr6:71924206C>T	Remission	Minus 7	0	1280	0.000%	Minus 37	5	11108	0.045%
AML 239	chr8:102855596A>G	Remission	Minus 7	0	1155	0.000%	Minus 37	26	10709	0.243%
AML 239	chr8:5679049T>A	Remission	Minus 7	2	1069	0.187%	Minus 37	18	10444	0.172%
AML 239	chr8:98557144G>A	Remission	Minus 7	1	1491	0.067%	Minus 37	7	11134	0.063%
AML 239	chr9:9417477C>T	Remission	Minus 7	5	1374	0.364%	Minus 37	11	9168	0.120%
AML 239	chrX:145090722C>G	Remission	Minus 7	4	1340	0.299%	Minus 37	13	8214	0.158%
AML 240	chr1:109547908GGCT>-	Remission	Minus 7	2	12715	0.016%	Minus 33	0	4664	0.000%
AML 240	chr1:210812092C>T	Remission	Minus 7	6	21544	0.028%	Minus 33	0	2970	0.000%
AML 240	chr1:28812945A>G	Remission	Minus 7	4	18600	0.022%	Minus 33	0	7524	0.000%
AML 240	chr10:116848175G>A	Remission	Minus 7	1	28523	0.004%	Minus 33	0	4559	0.000%
AML 240	chr10:13001010A>G	Remission	Minus 7	1	25956	0.004%	Minus 33	0	6485	0.000%
AML 240	chr10:33978321A>T	Remission	Minus 7	6	19842	0.030%	Minus 33	0	3196	0.000%
AML 240	chr10:97571292C>T	Remission	Minus 7	7	28710	0.024%	Minus 33	0	6828	0.000%
AML 240	chr11:127739135G>A	Remission	Minus 7	4	21106	0.019%	Minus 33	0	2862	0.000%
AML 240	chr11:33802572C>T	Remission	Minus 7	1	14303	0.007%	Minus 33	2	5874	0.034%
AML 240	chr11:64766111GCGGGA>-	Remission	Minus 7	3	18043	0.017%	Minus 33	1	9619	0.010%
AML 240	chr11:83954495A>-	Remission	Minus 7	4	23855	0.017%	Minus 33	0	1994	0.000%
AML 240	chr11:92213273C>T	Remission	Minus 7	2	21433	0.009%	Minus 33	0	3486	0.000%
AML 240	chr12:106669384A>G	Remission	Minus 7	5	27806	0.018%	Minus 33	0	5179	0.000%
AML 240	chr12:115470541C>T	Remission	Minus 7	1	22933	0.004%	Minus 33	1	5335	0.019%
AML 240	chr12:15164481C>T	Remission	Minus 7	10	28817	0.035%	Minus 33	1	4418	0.023%
AML 240	chr13:101946254A>C	Remission	Minus 7	6	23077	0.026%	Minus 33	0	4033	0.000%
AML 240	chr13:104750573G>A	Remission	Minus 7	4	21390	0.019%	Minus 33	0	3722	0.000%
AML 240	chr13:32961338C>T	Remission	Minus 7	6	24491	0.024%	Minus 33	0	3581	0.000%
AML 240	chr13:60030523C>T	Remission	Minus 7	3	23229	0.013%	Minus 33	1	2205	0.045%
AML 240	chr13:73371923C>T	Remission	Minus 7	4	29050	0.014%	Minus 33	0	4701	0.000%
AML 240	chr13:75764333C>T	Remission	Minus 7	3	24488	0.012%	Minus 33	0	3748	0.000%
AML 240	chr14:24871961C>T	Remission	Minus 7	4	20495	0.020%	Minus 33	0	3762	0.000%
AML 240	chr14:26199812G>A	Remission	Minus 7	6	24707	0.024%	Minus 33	0	4408	0.000%
AML 240	chr14:50861796G>A	Remission	Minus 7	6	27979	0.021%	Minus 33	0	4386	0.000%
AML 240	chr14:63420489T>C	Remission	Minus 7	2	21455	0.009%	Minus 33	0	3433	0.000%
AML 240	chr14:69702837T>C	Remission	Minus 7	2	22798	0.009%	Minus 33	0	3849	0.000%
AML 240	chr15:46572302A>T	Remission	Minus 7	5	20877	0.024%	Minus 33	0	5204	0.000%
AML 240	chr16:35393916G>A	Remission	Minus 7	6	29593	0.020%	Minus 33	0	5788	0.000%
AML 240	chr17:9987377T>C	Remission	Minus 7	6	25245	0.024%	Minus 33	1	5066	0.020%
AML 240	chr18:29787619A>G	Remission	Minus 7	8	22765	0.035%	Minus 33	0	3009	0.000%
AML 240	chr18:38657157T>C	Remission	Minus 7	5	24359	0.021%	Minus 33	0	3745	0.000%
AML 240	chr18:47225717G>C	Remission	Minus 7	1	23643	0.004%	Minus 33	1	5840	0.017%
AML 240	chr19:28501849G>A	Remission	Minus 7	4	19635	0.020%	Minus 33	3	4805	0.062%
AML 240	chr19:31907090A>G	Remission	Minus 7	5	24369	0.021%	Minus 33	0	4963	0.000%
AML 240	chr2:133680796A>T	Remission	Minus 7	6	14083	0.043%	Minus 33	0	2573	0.000%
AML 240	chr2:190251432C>G	Remission	Minus 7	7	24375	0.029%	Minus 33	0	3723	0.000%
AML 240	chr2:210937493T>C	Remission	Minus 7	5	23491	0.021%	Minus 33	0	3342	0.000%
AML 240	chr2:229057989C>T	Remission	Minus 7	2	23430	0.009%	Minus 33	0	3370	0.000%
AML 240	chr2:23320144G>C	Remission	Minus 7	2	24775	0.008%	Minus 33	1	5026	0.020%

AML 240	chr20:19049506G>C	Remission	Minus 7	1	16681	0.006%	Minus 33	0	4991	0.000%
AML 240	chr20:19868114T>C	Remission	Minus 7	7	23813	0.029%	Minus 33	2	7012	0.029%
AML 240	chr20:35945648G>A	Remission	Minus 7	1	30647	0.003%	Minus 33	0	6773	0.000%
AML 240	chr20:38836067A>G	Remission	Minus 7	6	24637	0.024%	Minus 33	0	9143	0.000%
AML 240	chr21:31807628G>A	Remission	Minus 7	7	29853	0.023%	Minus 33	1	5148	0.019%
AML 240	chr22:30743396->G	Remission	Minus 7	4	24129	0.017%	Minus 33	0	5601	0.000%
AML 240	chr3:59726355C>T	Remission	Minus 7	5	27261	0.018%	Minus 33	0	4681	0.000%
AML 240	chr4:172581470A>G	Remission	Minus 7	6	28012	0.021%	Minus 33	0	4782	0.000%
AML 240	chr4:72535542G>A	Remission	Minus 7	4	19806	0.020%	Minus 33	0	3127	0.000%
AML 240	chr5:120081430T>C	Remission	Minus 7	6	19239	0.031%	Minus 33	0	2042	0.000%
AML 240	chr5:36345987C>A	Remission	Minus 7	3	22655	0.013%	Minus 33	0	3719	0.000%
AML 240	chr5:4175162C>T	Remission	Minus 7	5	25935	0.019%	Minus 33	1	5663	0.018%
AML 240	chr6:110002085C>G	Remission	Minus 7	2	24900	0.008%	Minus 33	0	5041	0.000%
AML 240	chr6:148399671C>T	Remission	Minus 7	2	22086	0.009%	Minus 33	0	3775	0.000%
AML 240	chr7:25421764T>C	Remission	Minus 7	5	21007	0.024%	Minus 33	0	4038	0.000%
AML 240	chr7:5747470T>C	Remission	Minus 7	10	20119	0.050%	Minus 33	0	3633	0.000%
AML 240	chr8:104796591C>T	Remission	Minus 7	8	22540	0.035%	Minus 33	0	4022	0.000%
AML 240	chr8:2973560G>A	Remission	Minus 7	4	27599	0.014%	Minus 33	0	4384	0.000%
AML 240	chr8:34586152A>G	Remission	Minus 7	5	20540	0.024%	Minus 33	1	3351	0.030%
AML 240	chrX:108962525C>T	Remission	Minus 7	3	13202	0.023%	Minus 33	0	1880	0.000%
AML 240	chrX:134713782G>A	Remission	Minus 7	5	15245	0.033%	Minus 33	3	3537	0.085%
AML 241	chr1:155187917C>T	Remission	Minus 7	1	7418	0.013%	Minus 29	0	2452	0.000%
AML 241	chr1:167510196C>T	Remission	Minus 7	0	6082	0.000%	Minus 29	1	2514	0.040%
AML 241	chr1:77979302G>A	Remission	Minus 7	0	4296	0.000%	Minus 29	2	4093	0.049%
AML 241	chr1:77986583C>T	Remission	Minus 7	0	5524	0.000%	Minus 29	2	1488	0.134%
AML 241	chr10:117555749C>G	Remission	Minus 7	0	5823	0.000%	Minus 29	2	2781	0.072%
AML 241	chr10:14845383G>A	Remission	Minus 7	1	7060	0.014%	Minus 29	0	2128	0.000%
AML 241	chr10:69689216T>C	Remission	Minus 7	0	7342	0.000%	Minus 29	4	1796	0.223%
AML 241	chr10:7143027C>T	Remission	Minus 7	1	7419	0.013%	Minus 29	1	2519	0.040%
AML 241	chr10:98468128A>G	Remission	Minus 7	1	6512	0.015%	Minus 29	2	3757	0.053%
AML 241	chr11:115157549C>T	Remission	Minus 7	0	6395	0.000%	Minus 29	1	2621	0.038%
AML 241	chr11:121134967G>C	Remission	Minus 7	0	6936	0.000%	Minus 29	1	2194	0.046%
AML 241	chr11:130723284T>C	Remission	Minus 7	0	6998	0.000%	Minus 29	2	2208	0.091%
AML 241	chr11:42317219C>T	Remission	Minus 7	0	7235	0.000%	Minus 29	1	2923	0.034%
AML 241	chr11:61360611C>T	Remission	Minus 7	1	6535	0.015%	Minus 29	2	2610	0.077%
AML 241	chr13:42762113T>G	Remission	Minus 7	0	6851	0.000%	Minus 29	2	1786	0.112%
AML 241	chr14:103734851A>G	Remission	Minus 7	1	7299	0.014%	Minus 29	3	3510	0.085%
AML 241	chr15:40955538G>A	Remission	Minus 7	0	6321	0.000%	Minus 29	4	3352	0.119%
AML 241	chr15:63328667A>G	Remission	Minus 7	0	5760	0.000%	Minus 29	4	3417	0.117%
AML 241	chr15:87942979G>A	Remission	Minus 7	0	7529	0.000%	Minus 29	1	1948	0.051%
AML 241	chr16:50046356T>A	Remission	Minus 7	1	6283	0.016%	Minus 29	2	2718	0.074%
AML 241	chr16:56267079C>T	Remission	Minus 7	0	7487	0.000%	Minus 29	2	2336	0.086%
AML 241	chr16:65033077C>T	Remission	Minus 7	0	6474	0.000%	Minus 29	2	1175	0.170%
AML 241	chr16:67989741->A	Remission	Minus 7	1	7423	0.013%	Minus 29	0	2156	0.000%
AML 241	chr16:7397614T>G	Remission	Minus 7	0	5518	0.000%	Minus 29	1	954	0.105%
AML 241	chr16:89873831G>A	Remission	Minus 7	0	2283	0.000%	Minus 29	7	6920	0.101%
AML 241	chr2:75944816C>T	Remission	Minus 7	1	4924	0.020%	Minus 29	3	1537	0.195%
AML 241	chr20:2527620A>G	Remission	Minus 7	0	6526	0.000%	Minus 29	1	1731	0.058%
AML 241	chr20:50781999C>T	Remission	Minus 7	0	4392	0.000%	Minus 29	1	1767	0.057%
AML 241	chr21:31966916G>T	Remission	Minus 7	1	6368	0.016%	Minus 29	0	1453	0.000%

AML 241	chr3:100352613C>G	Remission	Minus 7	0	5934	0.000%	Minus 29	3	1721	0.174%
AML 241	chr3:25415772G>A	Remission	Minus 7	1	6190	0.016%	Minus 29	0	846	0.000%
AML 241	chr3:74439657C>T	Remission	Minus 7	1	6849	0.015%	Minus 29	0	1113	0.000%
AML 241	chr4:17013952C>T	Remission	Minus 7	0	7645	0.000%	Minus 29	2	1613	0.124%
AML 241	chr4:172813639G>C	Remission	Minus 7	1	7496	0.013%	Minus 29	0	1635	0.000%
AML 241	chr4:20946882A>C	Remission	Minus 7	0	4648	0.000%	Minus 29	1	872	0.115%
AML 241	chr4:24124142T>C	Remission	Minus 7	1	6248	0.016%	Minus 29	1	1787	0.056%
AML 241	chr4:40348954T>A	Remission	Minus 7	0	7629	0.000%	Minus 29	3	1906	0.157%
AML 241	chr4:73862187G>A	Remission	Minus 7	0	7458	0.000%	Minus 29	1	1664	0.060%
AML 241	chr5:42508532T>C	Remission	Minus 7	1	7419	0.013%	Minus 29	1	3934	0.025%
AML 241	chr5:7758962C>G	Remission	Minus 7	2	7607	0.026%	Minus 29	2	2168	0.092%
AML 241	chr6:118973072T>C	Remission	Minus 7	0	6337	0.000%	Minus 29	2	1388	0.144%
AML 241	chr6:11938765C>G	Remission	Minus 7	0	8491	0.000%	Minus 29	2	2320	0.086%
AML 241	chr6:134573972G>A	Remission	Minus 7	0	6854	0.000%	Minus 29	1	1262	0.079%
AML 241	chr6:148002316T>C	Remission	Minus 7	1	5384	0.019%	Minus 29	1	939	0.106%
AML 241	chr7:43550854T>C	Remission	Minus 7	0	6044	0.000%	Minus 29	2	1831	0.109%
AML 241	chr8:132863794->AATC	Remission	Minus 7	0	6101	0.000%	Minus 29	2	2531	0.079%
AML 241	chr8:137985582C>T	Remission	Minus 7	0	6637	0.000%	Minus 29	1	2119	0.047%
AML 241	chr9:11704606C>T	Remission	Minus 7	2	7776	0.026%	Minus 29	1	2782	0.036%
AML 241	chr9:125726985C>T	Remission	Minus 7	0	5846	0.000%	Minus 29	1	1036	0.097%
AML 241	chr9:610949G>C	Remission	Minus 7	0	7402	0.000%	Minus 29	2	1769	0.113%
AML 241	chr9:70944583G>A	Remission	Minus 7	0	6524	0.000%	Minus 29	3	1551	0.193%
AML 241	chrX:136907566G>A	Remission	Minus 7	0	2926	0.000%	Minus 29	2	846	0.236%
AML 241	chrX:29787252G>A	Remission	Minus 7	0	2997	0.000%	Minus 29	4	798	0.501%
AML 245	chr1:152627679C>T	Remission	Minus 7	4	1576	0.254%	Minus 106	1	4202	0.024%
AML 245	chr10:128489887A>T	Remission	Minus 7	4	2008	0.199%	Minus 106	4	6806	0.059%
AML 245	chr10:64219533A>G	Remission	Minus 7	3	1358	0.221%	Minus 106	1	4598	0.022%
AML 245	chr11:101016121A>G	Remission	Minus 7	6	1814	0.331%	Minus 106	4	4607	0.087%
AML 245	chr11:115142013G>A	Remission	Minus 7	1	1742	0.057%	Minus 106	3	7765	0.039%
AML 245	chr11:134426026C>T	Remission	Minus 7	5	1443	0.347%	Minus 106	2	6358	0.031%
AML 245	chr11:23631913T>A	Remission	Minus 7	3	1504	0.199%	Minus 106	0	3570	0.000%
AML 245	chr11:27580616C>T	Remission	Minus 7	4	1572	0.254%	Minus 106	2	5436	0.037%
AML 245	chr11:32396398G>-	Remission	Minus 7	4	1930	0.207%	Minus 106	0	7555	0.000%
AML 245	chr11:57304369G>A	Remission	Minus 7	0	1564	0.000%	Minus 106	4	8208	0.049%
AML 245	chr11:60890916C>A	Remission	Minus 7	2	1448	0.138%	Minus 106	5	6641	0.075%
AML 245	chr11:7763661G>A	Remission	Minus 7	7	1837	0.381%	Minus 106	1	3147	0.032%
AML 245	chr12:104348944T>C	Remission	Minus 7	4	1660	0.241%	Minus 106	1	5722	0.017%
AML 245	chr12:42857616T>G	Remission	Minus 7	0	1084	0.000%	Minus 106	2	3657	0.055%
AML 245	chr12:43511398C>T	Remission	Minus 7	7	1544	0.453%	Minus 106	1	5000	0.020%
AML 245	chr13:95797300G>A	Remission	Minus 7	2	1543	0.130%	Minus 106	1	3565	0.028%
AML 245	chr14:95613620G>A	Remission	Minus 7	0	1632	0.000%	Minus 106	2	5723	0.035%
AML 245	chr15:46780395C>G	Remission	Minus 7	2	1270	0.157%	Minus 106	2	4316	0.046%
AML 245	chr16:48789182C>G	Remission	Minus 7	5	1622	0.308%	Minus 106	3	6192	0.048%
AML 245	chr16:53732716ATAATA>-	Remission	Minus 7	4	1456	0.275%	Minus 106	0	5829	0.000%
AML 245	chr16:53947425A>G	Remission	Minus 7	5	1607	0.311%	Minus 106	1	4912	0.020%
AML 245	chr16:61077356T>A	Remission	Minus 7	5	1661	0.301%	Minus 106	1	3883	0.026%
AML 245	chr16:63573629A>G	Remission	Minus 7	3	1459	0.206%	Minus 106	1	3226	0.031%
AML 245	chr16:65028184C>T	Remission	Minus 7	5	1545	0.324%	Minus 106	3	5977	0.050%
AML 245	chr16:66199465C>T	Remission	Minus 7	2	531	0.377%	Minus 106	2	4667	0.043%
AML 245	chr16:70691086G>C	Remission	Minus 7	4	1851	0.216%	Minus 106	2	6925	0.029%

AML 245	chr17:69907658C>-	Remission	Minus 7	2	849	0.236%	Minus 106	4	5647	0.071%
AML 245	chr18:53489923A>G	Remission	Minus 7	5	1201	0.416%	Minus 106	0	3827	0.000%
AML 245	chr19:49148410C>T	Remission	Minus 7	2	1629	0.123%	Minus 106	2	5906	0.034%
AML 245	chr2:104124163A>T	Remission	Minus 7	6	1621	0.370%	Minus 106	1	3814	0.026%
AML 245	chr2:124516180T>C	Remission	Minus 7	7	1583	0.442%	Minus 106	2	4230	0.047%
AML 245	chr2:211282603G>C	Remission	Minus 7	4	1640	0.244%	Minus 106	1	3950	0.025%
AML 245	chr2:229170031C>T	Remission	Minus 7	2	1609	0.124%	Minus 106	3	6555	0.046%
AML 245	chr2:237737312TT>-	Remission	Minus 7	5	1812	0.276%	Minus 106	3	7500	0.040%
AML 245	chr2:27139588T>A	Remission	Minus 7	4	1679	0.238%	Minus 106	8	6123	0.131%
AML 245	chr2:51368616C>G	Remission	Minus 7	4	1510	0.265%	Minus 106	2	4024	0.050%
AML 245	chr20:20833493G>A	Remission	Minus 7	5	1909	0.262%	Minus 106	2	7715	0.026%
AML 245	chr20:396185C>G	Remission	Minus 7	4	1207	0.331%	Minus 106	1	4969	0.020%
AML 245	chr20:57951388G>C	Remission	Minus 7	5	1128	0.443%	Minus 106	4	6405	0.062%
AML 245	chr3:116051378T>C	Remission	Minus 7	2	1518	0.132%	Minus 106	2	4766	0.042%
AML 245	chr3:140493450A>G	Remission	Minus 7	7	1423	0.492%	Minus 106	0	4013	0.000%
AML 245	chr3:15070588C>G	Remission	Minus 7	3	1530	0.196%	Minus 106	1	4924	0.020%
AML 245	chr3:85935791C>T	Remission	Minus 7	0	1151	0.000%	Minus 106	2	2515	0.080%
AML 245	chr3:9808903T>C	Remission	Minus 7	6	1524	0.394%	Minus 106	4	6662	0.060%
AML 245	chr4:107434165T>G	Remission	Minus 7	2	1756	0.114%	Minus 106	3	4070	0.074%
AML 245	chr4:119684667T>C	Remission	Minus 7	3	1349	0.222%	Minus 106	0	4465	0.000%
AML 245	chr4:13280547C>T	Remission	Minus 7	2	1548	0.129%	Minus 106	1	6585	0.015%
AML 245	chr4:138023841C>T	Remission	Minus 7	5	1407	0.355%	Minus 106	0	3557	0.000%
AML 245	chr4:49002802G>A	Remission	Minus 7	5	1562	0.320%	Minus 106	2	4899	0.041%
AML 245	chr4:666408G>A	Remission	Minus 7	6	1479	0.406%	Minus 106	5	8621	0.058%
AML 245	chr4:94055532C>T	Remission	Minus 7	6	1499	0.400%	Minus 106	0	4851	0.000%
AML 245	chr5:140005751T>C	Remission	Minus 7	7	1830	0.383%	Minus 106	2	6954	0.029%
AML 245	chr5:153394254T>C	Remission	Minus 7	6	1378	0.435%	Minus 106	3	4278	0.070%
AML 245	chr5:16077151T>G	Remission	Minus 7	1	1737	0.058%	Minus 106	1	3951	0.025%
AML 245	chr5:68736259T>C	Remission	Minus 7	2	1923	0.104%	Minus 106	3	6906	0.043%
AML 245	chr5:75804472C>-	Remission	Minus 7	6	1578	0.380%	Minus 106	0	5067	0.000%
AML 245	chr6:7161727C>T	Remission	Minus 7	6	1711	0.351%	Minus 106	3	6137	0.049%
AML 245	chr6:76697773T>C	Remission	Minus 7	2	1157	0.173%	Minus 106	2	3534	0.057%
AML 245	chr6:9343408G>A	Remission	Minus 7	6	1549	0.387%	Minus 106	4	6710	0.060%
AML 245	chr7:121676280C>T	Remission	Minus 7	3	1494	0.201%	Minus 106	2	4674	0.043%
AML 245	chr7:156002360C>T	Remission	Minus 7	1	1829	0.055%	Minus 106	2	6466	0.031%
AML 245	chr7:28631844G>A	Remission	Minus 7	7	1548	0.452%	Minus 106	0	7569	0.000%
AML 245	chr7:68856218A>-	Remission	Minus 7	6	1263	0.475%	Minus 106	2	3883	0.052%
AML 245	chr8:122848836G>A	Remission	Minus 7	7	1699	0.412%	Minus 106	1	8800	0.011%
AML 245	chr8:139021656T>C	Remission	Minus 7	2	1594	0.125%	Minus 106	1	5097	0.020%
AML 245	chr8:53174237C>T	Remission	Minus 7	4	1425	0.281%	Minus 106	2	5337	0.037%
AML 245	chr8:55269024C>T	Remission	Minus 7	4	1346	0.297%	Minus 106	0	3580	0.000%
AML 245	chr9:117099481A>T	Remission	Minus 7	5	1519	0.329%	Minus 106	0	4133	0.000%
AML 245	chr9:86116555T>-	Remission	Minus 7	2	1932	0.104%	Minus 106	3	6811	0.044%
AML 245	chrX:11582347A>G	Remission	Minus 7	6	811	0.740%	Minus 106	5	2110	0.237%
AML 246	chr1:116694332G>A	Remission	Minus 7	0	6023	0.000%	Minus 65	3	6824	0.044%
AML 246	chr1:203507528G>A	Remission	Minus 7	0	3666	0.000%	Minus 65	2	7957	0.025%
AML 246	chr1:217913065G>A	Remission	Minus 7	0	5789	0.000%	Minus 65	1	5498	0.018%
AML 246	chr1:228108394C>A	Remission	Minus 7	0	4047	0.000%	Minus 65	1	7580	0.013%
AML 246	chr1:228816128C>T	Remission	Minus 7	0	5187	0.000%	Minus 65	1	5302	0.019%
AML 246	chr1:36661813C>T	Remission	Minus 7	1	4602	0.022%	Minus 65	2	5360	0.037%

AML 246	chr1:5713062G>A	Remission	Minus 7	1	5596	0.018%	Minus 65	3	7212	0.042%
AML 246	chr1:95368304G>A	Remission	Minus 7	1	4618	0.022%	Minus 65	0	5278	0.000%
AML 246	chr11:131422364C>T	Remission	Minus 7	0	5315	0.000%	Minus 65	3	6255	0.048%
AML 246	chr11:74574651C>T	Remission	Minus 7	0	3830	0.000%	Minus 65	1	4820	0.021%
AML 246	chr11:87718570A>T	Remission	Minus 7	0	3731	0.000%	Minus 65	2	4316	0.046%
AML 246	chr11:9087665C>T	Remission	Minus 7	0	4412	0.000%	Minus 65	3	6011	0.050%
AML 246	chr11:92984341C>T	Remission	Minus 7	1	4807	0.021%	Minus 65	1	4892	0.020%
AML 246	chr12:106579286C>T	Remission	Minus 7	1	5036	0.020%	Minus 65	3	6740	0.045%
AML 246	chr13:101079503C>T	Remission	Minus 7	0	5334	0.000%	Minus 65	3	4615	0.065%
AML 246	chr13:49537117A>T	Remission	Minus 7	0	4153	0.000%	Minus 65	2	4297	0.047%
AML 246	chr15:100374022C>T	Remission	Minus 7	0	2958	0.000%	Minus 65	4	6595	0.061%
AML 246	chr15:101307901C>T	Remission	Minus 7	1	4024	0.025%	Minus 65	3	2757	0.109%
AML 246	chr15:93674127G>A	Remission	Minus 7	1	4503	0.022%	Minus 65	2	6509	0.031%
AML 246	chr15:96286146G>A	Remission	Minus 7	1	5376	0.019%	Minus 65	2	5890	0.034%
AML 246	chr16:27521585C>T	Remission	Minus 7	0	5162	0.000%	Minus 65	1	7306	0.014%
AML 246	chr16:30513071G>A	Remission	Minus 7	2	5419	0.037%	Minus 65	1	6329	0.016%
AML 246	chr16:4831053A>T	Remission	Minus 7	0	3682	0.000%	Minus 65	3	5686	0.053%
AML 246	chr16:52601311T>C	Remission	Minus 7	0	3641	0.000%	Minus 65	2	4471	0.045%
AML 246	chr16:62009465T>C	Remission	Minus 7	0	4363	0.000%	Minus 65	1	4776	0.021%
AML 246	chr16:66245301C>T	Remission	Minus 7	0	4986	0.000%	Minus 65	4	7406	0.054%
AML 246	chr16:66292578C>T	Remission	Minus 7	1	4713	0.021%	Minus 65	0	7089	0.000%
AML 246	chr17:17465118G>A	Remission	Minus 7	0	5152	0.000%	Minus 65	1	5796	0.017%
AML 246	chr17:33815526G>A	Remission	Minus 7	0	4933	0.000%	Minus 65	1	6394	0.016%
AML 246	chr17:7675124T>C	Remission	Minus 7	0	5420	0.000%	Minus 65	2	9169	0.022%
AML 246	hr17:79394816CGTCCCGGGC	Remission	Minus 7	0	4301	0.000%	Minus 65	1	7058	0.014%
AML 246	chr18:32912067G>A	Remission	Minus 7	0	6312	0.000%	Minus 65	1	5872	0.017%
AML 246	chr18:63295797C>T	Remission	Minus 7	0	5417	0.000%	Minus 65	1	6071	0.016%
AML 246	chr2:133732853C>G	Remission	Minus 7	1	5516	0.018%	Minus 65	1	4675	0.021%
AML 246	chr2:191837512C>T	Remission	Minus 7	1	4694	0.021%	Minus 65	0	6654	0.000%
AML 246	chr2:201778150C>T	Remission	Minus 7	0	4012	0.000%	Minus 65	1	4718	0.021%
AML 246	chr2:57406280G>A	Remission	Minus 7	0	4073	0.000%	Minus 65	2	4808	0.042%
AML 246	chr2:81621992C>A	Remission	Minus 7	0	3727	0.000%	Minus 65	1	4585	0.022%
AML 246	chr3:122980124C>T	Remission	Minus 7	0	5650	0.000%	Minus 65	2	6859	0.029%
AML 246	chr3:13665878C>T	Remission	Minus 7	0	5773	0.000%	Minus 65	1	9553	0.010%
AML 246	chr4:153936336G>A	Remission	Minus 7	0	4354	0.000%	Minus 65	2	5720	0.035%
AML 246	chr7:132547631G>A	Remission	Minus 7	1	5676	0.018%	Minus 65	0	7594	0.000%
AML 246	chr9:107074661C>A	Remission	Minus 7	0	4336	0.000%	Minus 65	2	4984	0.040%
AML 246	chr9:138075085G>A	Remission	Minus 7	0	4387	0.000%	Minus 65	1	5428	0.018%
AML 246	chr9:28394211T>A	Remission	Minus 7	1	4283	0.023%	Minus 65	2	4492	0.045%
AML 246	chr9:70665332C>T	Remission	Minus 7	0	4586	0.000%	Minus 65	2	4068	0.049%
AML 246	chr9:91843707G>A	Remission	Minus 7	0	5685	0.000%	Minus 65	1	7756	0.013%
AML 246	chrX:132804311C>G	Remission	Minus 7	0	3063	0.000%	Minus 65	4	3561	0.112%
AML 246	chrX:141217201T>A	Remission	Minus 7	0	2586	0.000%	Minus 65	2	3073	0.065%
AML 246	chrX:17170935CCT>-	Remission	Minus 7	1	2598	0.038%	Minus 65	0	2730	0.000%
AML 246	chrX:53217773C>A	Remission	Minus 7	0	2603	0.000%	Minus 65	2	3533	0.057%
AML 248	chr1:14609249G>A	Remission	Minus 7	121	21032	0.575%	Minus 16	39	10313	0.378%
AML 248	chr1:147656369C>T	Remission	Minus 7	278	22173	1.254%	Minus 16	66	7668	0.861%
AML 248	chr1:203737152A>G	Remission	Minus 7	185	16969	1.090%	Minus 16	36	4145	0.869%
AML 248	chr1:2820056G>A	Remission	Minus 7	53	22852	0.232%	Minus 16	6	12583	0.048%
AML 248	chr1:40866191T>C	Remission	Minus 7	552	23906	2.309%	Minus 16	229	11033	2.076%

AML 248	chr1:7466894C>T	Remission	Minus 7	48	25539	0.188%	Minus 16	15	12228	0.123%
AML 248	chr10:118507409T>C	Remission	Minus 7	48	19785	0.243%	Minus 16	7	9126	0.077%
AML 248	chr10:16313939T>A	Remission	Minus 7	54	18361	0.294%	Minus 16	6	8097	0.074%
AML 248	chr10:17429036->A	Remission	Minus 7	1	12801	0.008%	Minus 16	0	9575	0.000%
AML 248	chr10:70267661C>T	Remission	Minus 7	3	20942	0.014%	Minus 16	0	10402	0.000%
AML 248	chr11:11641040G>A	Remission	Minus 7	53	22908	0.231%	Minus 16	9	10830	0.083%
AML 248	ır11:134654301->GGGTGCAT	Remission	Minus 7	176	21530	0.817%	Minus 16	71	11502	0.617%
AML 248	chr11:36861290G>A	Remission	Minus 7	576	16638	3.462%	Minus 16	189	6111	3.093%
AML 248	chr11:94722188G>A	Remission	Minus 7	57	19410	0.294%	Minus 16	6	8100	0.074%
AML 248	chr12:130171084A>G	Remission	Minus 7	0	23482	0.000%	Minus 16	2	9832	0.020%
AML 248	chr13:104615517G>A	Remission	Minus 7	0	17521	0.000%	Minus 16	2	7274	0.027%
AML 248	chr13:21126550C>T	Remission	Minus 7	1	24103	0.004%	Minus 16	0	10454	0.000%
AML 248	chr13:48117971A>G	Remission	Minus 7	111	24808	0.447%	Minus 16	21	10039	0.209%
AML 248	chr13:52962844A>G	Remission	Minus 7	1	18438	0.005%	Minus 16	3	8260	0.036%
AML 248	chr13:62224453C>T	Remission	Minus 7	48	17767	0.270%	Minus 16	2	6443	0.031%
AML 248	chr13:93287606G>A	Remission	Minus 7	44	15200	0.289%	Minus 16	3	6104	0.049%
AML 248	chr14:39119285G>A	Remission	Minus 7	117	19849	0.589%	Minus 16	23	5652	0.407%
AML 248	chr14:95340582G>A	Remission	Minus 7	3	25745	0.012%	Minus 16	0	12301	0.000%
AML 248	chr15:37780604C>T	Remission	Minus 7	0	25758	0.000%	Minus 16	3	9040	0.033%
AML 248	chr15:85938083C>T	Remission	Minus 7	57	21828	0.261%	Minus 16	12	9781	0.123%
AML 248	chr16:51684669A>G	Remission	Minus 7	2	23467	0.009%	Minus 16	1	10494	0.010%
AML 248	chr16:86772840G>C	Remission	Minus 7	0	22087	0.000%	Minus 16	3	11160	0.027%
AML 248	chr16:9871241->A	Remission	Minus 7	41	16804	0.244%	Minus 16	3	10639	0.028%
AML 248	chr17:5766396T>C	Remission	Minus 7	67	28461	0.235%	Minus 16	16	12970	0.123%
AML 248	chr17:70021736C>A	Remission	Minus 7	3	19140	0.016%	Minus 16	0	7601	0.000%
AML 248	chr19:19096041C>G	Remission	Minus 7	173	25863	0.669%	Minus 16	55	14163	0.388%
AML 248	chr19:33701142C>T	Remission	Minus 7	58	27608	0.210%	Minus 16	18	13416	0.134%
AML 248	chr2:158126080G>A	Remission	Minus 7	55	18469	0.298%	Minus 16	14	8446	0.166%
AML 248	chr2:18197156G>A	Remission	Minus 7	292	16260	1.796%	Minus 16	92	6997	1.315%
AML 248	chr2:220747088C>G	Remission	Minus 7	1	14557	0.007%	Minus 16	0	6258	0.000%
AML 248	chr2:221271014A>T	Remission	Minus 7	647	17939	3.607%	Minus 16	298	7513	3.966%
AML 248	chr2:239881851A>G	Remission	Minus 7	80	23354	0.343%	Minus 16	9	12381	0.073%
AML 248	chr2:3974847C>T	Remission	Minus 7	49	22321	0.220%	Minus 16	12	8685	0.138%
AML 248	chr2:49176542T>C	Remission	Minus 7	651	20531	3.171%	Minus 16	250	8999	2.778%
AML 248	chr2:66358307C>T	Remission	Minus 7	43	17365	0.248%	Minus 16	4	5614	0.071%
AML 248	chr2:72986641A>G	Remission	Minus 7	0	20148	0.000%	Minus 16	2	10114	0.020%
AML 248	chr2:9414541C>T	Remission	Minus 7	66	23009	0.287%	Minus 16	12	9476	0.127%
AML 248	chr20:61123120C>T	Remission	Minus 7	0	17243	0.000%	Minus 16	4	9297	0.043%
AML 248	chr21:25343636C>T	Remission	Minus 7	57	20286	0.281%	Minus 16	10	8122	0.123%
AML 248	chr21:37677112C>T	Remission	Minus 7	380	20929	1.816%	Minus 16	140	11775	1.189%
AML 248	chr22:33909039C>T	Remission	Minus 7	46	22502	0.204%	Minus 16	6	9000	0.067%
AML 248	chr22:47378266G>A	Remission	Minus 7	234	19948	1.173%	Minus 16	107	10578	1.012%
AML 248	chr3:105395780G>C	Remission	Minus 7	44	16943	0.260%	Minus 16	3	6216	0.048%
AML 248	chr3:138365619T>C	Remission	Minus 7	58	22033	0.263%	Minus 16	7	10789	0.065%
AML 248	chr3:144253083A>G	Remission	Minus 7	0	19951	0.000%	Minus 16	2	7213	0.028%
AML 248	chr3:15268647T>C	Remission	Minus 7	78	26165	0.298%	Minus 16	5	11570	0.043%
AML 248	chr3:155069427G>T	Remission	Minus 7	463	21631	2.140%	Minus 16	147	7138	2.059%
AML 248	chr3:193344044G>T	Remission	Minus 7	565	23678	2.386%	Minus 16	170	9229	1.842%
AML 248	chr3:36719449C>T	Remission	Minus 7	49	19841	0.247%	Minus 16	3	7557	0.040%
AML 248	chr4:114087248T>C	Remission	Minus 7	19	6433	0.295%	Minus 16	3	1480	0.203%

AML 248	chr5:117359851G>A	Remission	Minus 7	0	14669	0.000%	Minus 16	3	6320	0.047%
AML 248	chr5:138420903C>T	Remission	Minus 7	71	23078	0.308%	Minus 16	11	10798	0.102%
AML 248	chr5:37768102G>C	Remission	Minus 7	260	23785	1.093%	Minus 16	83	10151	0.818%
AML 248	chr5:38936499G>-	Remission	Minus 7	652	21759	2.996%	Minus 16	199	8323	2.391%
AML 248	chr5:43420421C>T	Remission	Minus 7	1	19488	0.005%	Minus 16	0	7257	0.000%
AML 248	chr5:5942386C>T	Remission	Minus 7	46	20134	0.228%	Minus 16	9	8454	0.106%
AML 248	chr5:64188253A>T	Remission	Minus 7	6	17917	0.033%	Minus 16	3	6319	0.047%
AML 248	chr5:80012326G>A	Remission	Minus 7	2	19677	0.010%	Minus 16	2	8407	0.024%
AML 248	chr6:105590513G>A	Remission	Minus 7	1	21903	0.005%	Minus 16	2	9062	0.022%
AML 248	chr6:109374931A>G	Remission	Minus 7	1	17964	0.006%	Minus 16	2	7128	0.028%
AML 248	chr6:78163810G>A	Remission	Minus 7	1	10684	0.009%	Minus 16	1	3425	0.029%
AML 248	chr6:84798255C>A	Remission	Minus 7	90	27774	0.324%	Minus 16	10	8919	0.112%
AML 248	chr6:89931274C>G	Remission	Minus 7	0	19148	0.000%	Minus 16	1	6878	0.015%
AML 248	chr7:118695138C>T	Remission	Minus 7	235	17544	1.339%	Minus 16	66	6061	1.089%
AML 248	chr7:132095315C>T	Remission	Minus 7	191	19196	0.995%	Minus 16	64	8618	0.743%
AML 248	chr7:132300844G>A	Remission	Minus 7	47	20958	0.224%	Minus 16	8	12034	0.066%
AML 248	chr7:158236471C>T	Remission	Minus 7	172	25683	0.670%	Minus 16	61	12242	0.498%
AML 248	chr7:8418476G>A	Remission	Minus 7	56	19728	0.284%	Minus 16	4	5679	0.070%
AML 248	chr8:139131792G>A	Remission	Minus 7	33	14715	0.224%	Minus 16	10	7507	0.133%
AML 248	chr8:32518458T>C	Remission	Minus 7	525	16814	3.122%	Minus 16	201	6980	2.880%
AML 248	chr8:3460641T>C	Remission	Minus 7	182	16080	1.132%	Minus 16	49	5840	0.839%
AML 248	chr8:41719460G>A	Remission	Minus 7	78	23704	0.329%	Minus 16	14	13034	0.107%
AML 248	chr8:60062081G>C	Remission	Minus 7	49	21128	0.232%	Minus 16	4	6733	0.059%
AML 248	chr9:132157050C>T	Remission	Minus 7	256	21486	1.191%	Minus 16	95	10774	0.882%
AML 248	chrX:17613357G>A	Remission	Minus 7	5	13537	0.037%	Minus 16	5	5547	0.090%
AML 250	chr1:181789617G>A	Remission	Minus 7	1	10253	0.010%	Minus 92	0	9823	0.000%
AML 250	chr1:214010845G>C	Remission	Minus 7	3	8744	0.034%	Minus 92	0	9968	0.000%
AML 250	chr1:221501136T>C	Remission	Minus 7	5	7986	0.063%	Minus 92	11	6923	0.159%
AML 250	chr1:237282606T>C	Remission	Minus 7	1	7323	0.014%	Minus 92	0	8436	0.000%
AML 250	chr1:23924096A>G	Remission	Minus 7	3	6706	0.045%	Minus 92	1	4760	0.021%
AML 250	chr10:89310721G>A	Remission	Minus 7	2	8706	0.023%	Minus 92	2	8739	0.023%
AML 250	chr11:131996720C>T	Remission	Minus 7	0	10096	0.000%	Minus 92	2	10240	0.020%
AML 250	chr12:129949400C>T	Remission	Minus 7	4	9367	0.043%	Minus 92	1	10226	0.010%
AML 250	chr12:70325021T>G	Remission	Minus 7	2	6953	0.029%	Minus 92	0	8193	0.000%
AML 250	chr12:77247619T>G	Remission	Minus 7	6	11998	0.050%	Minus 92	3	11847	0.025%
AML 250	chr16:12095420T>C	Remission	Minus 7	1	4017	0.025%	Minus 92	6	8557	0.070%
AML 250	chr16:48954956C>G	Remission	Minus 7	5	9286	0.054%	Minus 92	4	8788	0.046%
AML 250	chr16:6012946G>T	Remission	Minus 7	0	6941	0.000%	Minus 92	1	6793	0.015%
AML 250	chr17:530811C>T	Remission	Minus 7	1	8407	0.012%	Minus 92	4	9224	0.043%
AML 250	chr17:61306084A>G	Remission	Minus 7	1	7758	0.013%	Minus 92	2	8801	0.023%
AML 250	chr17:63631651T>-	Remission	Minus 7	0	5662	0.000%	Minus 92	1	2751	0.036%
AML 250	chr17:65994202C>T	Remission	Minus 7	1	9878	0.010%	Minus 92	2	10546	0.019%
AML 250	chr17:66238210G>A	Remission	Minus 7	4	10409	0.038%	Minus 92	3	9639	0.031%
AML 250	chr17:79808688C>T	Remission	Minus 7	2	11044	0.018%	Minus 92	2	9053	0.022%
AML 250	chr19:21590627G>A	Remission	Minus 7	3	8833	0.034%	Minus 92	1	9157	0.011%
AML 250	chr2:174094079G>A	Remission	Minus 7	0	10479	0.000%	Minus 92	1	9449	0.011%
AML 250	chr2:50581734G>A	Remission	Minus 7	0	8822	0.000%	Minus 92	2	8458	0.024%
AML 250	chr2:7813343C>T	Remission	Minus 7	3	11492	0.026%	Minus 92	2	11236	0.018%
AML 250	chr20:51346180C>-	Remission	Minus 7	2	11209	0.018%	Minus 92	3	10491	0.029%
AML 250	chr20:58750265C>T	Remission	Minus 7	3	10300	0.029%	Minus 92	0	10600	0.000%

AML 250	chr3:74698594C>A	Remission	Minus 7	1	6542	0.015%	Minus 92	1	6817	0.015%
AML 250	chr4:160661295T>C	Remission	Minus 7	0	7841	0.000%	Minus 92	1	7126	0.014%
AML 250	chr4:76710373G>A	Remission	Minus 7	3	8842	0.034%	Minus 92	0	12242	0.000%
AML 250	chr5:110274545C>T	Remission	Minus 7	1	9841	0.010%	Minus 92	0	8370	0.000%
AML 250	chr5:120940492C>-	Remission	Minus 7	3	6462	0.046%	Minus 92	0	7966	0.000%
AML 250	chr5:138657393C>G	Remission	Minus 7	4	11137	0.036%	Minus 92	2	10216	0.020%
AML 250	chr5:174502164T>G	Remission	Minus 7	0	10336	0.000%	Minus 92	6	10902	0.055%
AML 250	chr5:20054573T>C	Remission	Minus 7	5	9889	0.051%	Minus 92	0	8529	0.000%
AML 250	chr5:93555256A>G	Remission	Minus 7	3	7616	0.039%	Minus 92	0	7243	0.000%
AML 250	chr6:12234805G>A	Remission	Minus 7	4	9579	0.042%	Minus 92	1	9472	0.011%
AML 250	chr6:72862557A>G	Remission	Minus 7	1	4768	0.021%	Minus 92	2	3830	0.052%
AML 250	chr7:118263231A>G	Remission	Minus 7	0	7086	0.000%	Minus 92	5	8503	0.059%
AML 250	chr7:128254555A>T	Remission	Minus 7	3	11599	0.026%	Minus 92	3	11992	0.025%
AML 250	chr7:135229656A>G	Remission	Minus 7	3	8458	0.035%	Minus 92	2	10181	0.020%
AML 250	chr7:86748341C>G	Remission	Minus 7	4	6740	0.059%	Minus 92	5	8331	0.060%
AML 250	chr7:88486002C>T	Remission	Minus 7	1	6867	0.015%	Minus 92	0	8910	0.000%
AML 250	chr8:10943046G>A	Remission	Minus 7	2	10307	0.019%	Minus 92	0	8279	0.000%
AML 250	chr8:124042625C>T	Remission	Minus 7	0	8193	0.000%	Minus 92	1	7818	0.013%
AML 250	chr8:30743661G>C	Remission	Minus 7	4	7899	0.051%	Minus 92	4	8986	0.045%
AML 250	chr8:69333573C>G	Remission	Minus 7	2	8433	0.024%	Minus 92	2	10084	0.020%
AML 250	chr9:24310680A>C	Remission	Minus 7	2	6236	0.032%	Minus 92	3	4618	0.065%
AML 250	chrX:101261008C>T	Remission	Minus 7	1	9622	0.010%	Minus 92	0	11346	0.000%
AML 250	chrX:103772364T>C	Remission	Minus 7	1	10061	0.010%	Minus 92	1	8986	0.011%
AML 250	chrX:79618247C>T	Remission	Minus 7	2	9033	0.022%	Minus 92	0	10713	0.000%
AML 251	chr1:113913185G>C	Remission	Minus 7	29	32235	0.090%	Minus 38	0	8407	0.000%
AML 251	chr1:155904480T>A	Remission	Minus 7	30	31612	0.095%	Minus 38	4	6966	0.057%
AML 251	chr1:179669423G>C	Remission	Minus 7	22	35783	0.061%	Minus 38	0	7675	0.000%
AML 251	chr1:223462447G>C	Remission	Minus 7	11	26649	0.041%	Minus 38	1	6194	0.016%
AML 251	chr1:75025278C>G	Remission	Minus 7	10	29112	0.034%	Minus 38	3	4331	0.069%
AML 251	chr10:106665258G>C	Remission	Minus 7	24	38284	0.063%	Minus 38	0	5845	0.000%
AML 251	chr10:36621510G>C	Remission	Minus 7	12	30767	0.039%	Minus 38	0	5306	0.000%
AML 251	chr10:82944086->GGGAC	Remission	Minus 7	2	13749	0.015%	Minus 38	0	3030	0.000%
AML 251	chr11:100254069A>T	Remission	Minus 7	9	14816	0.061%	Minus 38	0	3331	0.000%
AML 251	chr11:102346774G>T	Remission	Minus 7	1	24935	0.004%	Minus 38	0	7475	0.000%
AML 251	chr11:123643117C>G	Remission	Minus 7	30	27414	0.109%	Minus 38	0	8904	0.000%
AML 251	chr11:5515469G>C	Remission	Minus 7	2	36336	0.006%	Minus 38	0	8059	0.000%
AML 251	chr11:81220644G>A	Remission	Minus 7	16	32851	0.049%	Minus 38	0	6809	0.000%
AML 251	chr11:99020392T>C	Remission	Minus 7	27	30712	0.088%	Minus 38	1	7024	0.014%
AML 251	chr12:115262331G>-	Remission	Minus 7	26	42581	0.061%	Minus 38	3	7440	0.040%
AML 251	chr12:127227392G>C	Remission	Minus 7	5	25313	0.020%	Minus 38	0	4476	0.000%
AML 251	chr12:49052371T>C	Remission	Minus 7	21	33591	0.063%	Minus 38	0	8738	0.000%
AML 251	chr12:65004477T>A	Remission	Minus 7	14	33764	0.041%	Minus 38	2	6966	0.029%
AML 251	chr12:85898023T>C	Remission	Minus 7	16	27599	0.058%	Minus 38	2	6406	0.031%
AML 251	chr12:93574960->GA	Remission	Minus 7	19	31791	0.060%	Minus 38	0	6245	0.000%
AML 251	chr12:97751063G>A	Remission	Minus 7	18	27874	0.065%	Minus 38	0	6135	0.000%
AML 251	chr13:65393072G>T	Remission	Minus 7	18	31644	0.057%	Minus 38	2	6278	0.032%
AML 251	chr13:85906764G>T	Remission	Minus 7	23	33138	0.069%	Minus 38	2	5133	0.039%
AML 251	chr13:96373712C>T	Remission	Minus 7	21	29569	0.071%	Minus 38	2	5358	0.037%
AML 251	chr14:29615725G>C	Remission	Minus 7	13	29467	0.044%	Minus 38	0	5577	0.000%
AML 251	chr15:101389694T>C	Remission	Minus 7	25	32344	0.077%	Minus 38	1	8421	0.012%

AML 251	chr15:86080219T>C	Remission	Minus 7	14	33922	0.041%	Minus 38	0	7081	0.000%
AML 251	chr16:64315580C>G	Remission	Minus 7	3	26480	0.011%	Minus 38	0	6085	0.000%
AML 251	chr17:30203376A>G	Remission	Minus 7	20	30256	0.066%	Minus 38	2	5944	0.034%
AML 251	chr18:11378989G>C	Remission	Minus 7	1	36982	0.003%	Minus 38	0	8452	0.000%
AML 251	chr18:54001774G>C	Remission	Minus 7	9	32277	0.028%	Minus 38	1	7181	0.014%
AML 251	chr18:60227705C>T	Remission	Minus 7	27	32804	0.082%	Minus 38	1	5816	0.017%
AML 251	chr18:70524437A>T	Remission	Minus 7	34	38943	0.087%	Minus 38	2	7061	0.028%
AML 251	chr19:33623896C>A	Remission	Minus 7	21	33715	0.062%	Minus 38	2	8469	0.024%
AML 251	chr19:57411010TTAAC>-	Remission	Minus 7	2	23875	0.008%	Minus 38	0	7813	0.000%
AML 251	chr2:103041103C>A	Remission	Minus 7	28	37043	0.076%	Minus 38	1	6408	0.016%
AML 251	chr2:173821810C>G	Remission	Minus 7	8	33042	0.024%	Minus 38	3	8571	0.035%
AML 251	chr2:20903741C>G	Remission	Minus 7	11	26802	0.041%	Minus 38	0	6734	0.000%
AML 251	chr2:212491338A>G	Remission	Minus 7	18	32485	0.055%	Minus 38	0	4044	0.000%
AML 251	chr2:239529022C>T	Remission	Minus 7	23	34866	0.066%	Minus 38	0	8640	0.000%
AML 251	chr2:30056856G>A	Remission	Minus 7	33	36314	0.091%	Minus 38	2	6380	0.031%
AML 251	chr2:80125435T>A	Remission	Minus 7	2	32234	0.006%	Minus 38	0	7004	0.000%
AML 251	chr20:40220782G>T	Remission	Minus 7	8	35882	0.022%	Minus 38	1	7823	0.013%
AML 251	chr21:14885818T>A	Remission	Minus 7	5	33093	0.015%	Minus 38	0	6415	0.000%
AML 251	chr21:46412475C>T	Remission	Minus 7	20	31713	0.063%	Minus 38	2	8224	0.024%
AML 251	chr22:46163735->CCGGT	Remission	Minus 7	6	28723	0.021%	Minus 38	0	8350	0.000%
AML 251	chr3:122121049G>A	Remission	Minus 7	21	36782	0.057%	Minus 38	2	6750	0.030%
AML 251	chr3:13190265->GCGCA	Remission	Minus 7	8	34257	0.023%	Minus 38	0	9171	0.000%
AML 251	chr3:181814957G>A	Remission	Minus 7	19	32665	0.058%	Minus 38	2	6778	0.030%
AML 251	chr3:193785856G>C	Remission	Minus 7	20	32009	0.062%	Minus 38	4	8754	0.046%
AML 251	chr3:2451012G>C	Remission	Minus 7	3	33822	0.009%	Minus 38	0	5979	0.000%
AML 251	chr3:48782120T>G	Remission	Minus 7	31	33733	0.092%	Minus 38	0	6266	0.000%
AML 251	chr3:85974475A>T	Remission	Minus 7	4	37948	0.011%	Minus 38	0	5906	0.000%
AML 251	chr4:108258111->AAG	Remission	Minus 7	1	11448	0.009%	Minus 38	0	2533	0.000%
AML 251	chr4:4944193G>T	Remission	Minus 7	14	14555	0.096%	Minus 38	2	4876	0.041%
AML 251	chr5:26460730A>T	Remission	Minus 7	1	31976	0.003%	Minus 38	0	6585	0.000%
AML 251	chr5:52223002T>C	Remission	Minus 7	14	27382	0.051%	Minus 38	0	3465	0.000%
AML 251	chr5:9783059G>A	Remission	Minus 7	24	31926	0.075%	Minus 38	1	7743	0.013%
AML 251	chr6:107663343G>A	Remission	Minus 7	30	40115	0.075%	Minus 38	0	7713	0.000%
AML 251	chr6:163301683T>C	Remission	Minus 7	38	41228	0.092%	Minus 38	0	7642	0.000%
AML 251	chr6:163336041->GGGGCA	Remission	Minus 7	13	29668	0.044%	Minus 38	3	8442	0.036%
AML 251	chr6:68956057G>C	Remission	Minus 7	17	34527	0.049%	Minus 38	0	8356	0.000%
AML 251	chr7:100561558C>G	Remission	Minus 7	28	39753	0.070%	Minus 38	2	8596	0.023%
AML 251	chr7:117041084G>C	Remission	Minus 7	21	26901	0.078%	Minus 38	1	4737	0.021%
AML 251	chr7:143951856G>A	Remission	Minus 7	19	35260	0.054%	Minus 38	0	8536	0.000%
AML 251	chr7:147509396T>C	Remission	Minus 7	15	32057	0.047%	Minus 38	1	5929	0.017%
AML 251	chr7:47605953->TGCCC	Remission	Minus 7	10	34779	0.029%	Minus 38	0	7938	0.000%
AML 251	chr8:102015064T>C	Remission	Minus 7	31	39753	0.078%	Minus 38	0	6695	0.000%
AML 251	chr8:14238270C>A	Remission	Minus 7	23	30409	0.076%	Minus 38	0	5047	0.000%
AML 251	chr8:32722302->TGA	Remission	Minus 7	12	29979	0.040%	Minus 38	0	4753	0.000%
AML 251	chr9:137746715C>T	Remission	Minus 7	7	30507	0.023%	Minus 38	1	5735	0.017%
AML 251	chr9:5231567T>A	Remission	Minus 7	33	34802	0.095%	Minus 38	0	9692	0.000%
AML 251	chrX:152907200G>T	Remission	Minus 7	2	37336	0.005%	Minus 38	0	10321	0.000%
AML 251	chrX:29072621->CCCC	Remission	Minus 7	9	34054	0.026%	Minus 38	0	5933	0.000%
AML 251	chrX:6231428T>A	Remission	Minus 7	23	34753	0.066%	Minus 38	1	7862	0.013%
AML 121	chr10:63867282G>A	3 months post-transplant	87	1	11534	0.009%	87	0	6796	0.000%

AML 121	chr13:113254106G>A	3 months post-transplant	87	1	12055	0.008%	87	0	6822	0.000%
AML 121	chr2:104729661C>G	3 months post-transplant	87	2	15999	0.013%	87	0	7920	0.000%
AML 121	chr9:68940919G>C	3 months post-transplant	87	0	10311	0.000%	87	1	6748	0.015%
AML 122	chr10:129942497C>G	3 months post-transplant	89	1	15251	0.007%	89	0	5088	0.000%
AML 122	chr11:28544552G>C	3 months post-transplant	89	7	6902	0.101%	89	4	3959	0.101%
AML 122	chr11:57934563A>G	3 months post-transplant	89	10	10996	0.091%	89	1	2547	0.039%
AML 122	chr12:124676438T>G	3 months post-transplant	89	3	11615	0.026%	89	4	4907	0.082%
AML 122	chr16:2598618C>A	3 months post-transplant	89	18	20012	0.090%	89	5	5186	0.096%
AML 122	chr18:49843886G>-	3 months post-transplant	89	1	3448	0.029%	89	0	4080	0.000%
AML 122	chr2:141283092T>C	3 months post-transplant	89	1	12448	0.008%	89	0	3009	0.000%
AML 122	chr2:25239199A>G	3 months post-transplant	89	8	13482	0.059%	89	2	3585	0.056%
AML 122	chr4:138104421A>C	3 months post-transplant	89	6	11278	0.053%	89	3	4927	0.061%
AML 122	chr7:122044719T>A	3 months post-transplant	89	14	22241	0.063%	89	2	4617	0.043%
AML 123	chr2:236010356G>C	3 months post-transplant	84	1	8073	0.012%	84	0	8875	0.000%
AML 123	chr7:148842182T>C	3 months post-transplant	84	1	167	0.599%	84	0	8720	0.000%
AML 123	chrX:134750569C>G	3 months post-transplant	84	1	4921	0.020%	84	0	4304	0.000%
AML 129	chr11:133840616A>G	3 months post-transplant	90	1	14660	0.007%	90	0	9937	0.000%
AML 129	chr15:23860086C>G	3 months post-transplant	90	1	14655	0.007%	90	0	11343	0.000%
AML 129	chr2:142221145A>G	3 months post-transplant	90	1	15810	0.006%	90	0	11785	0.000%
AML 129	chr20:7940367A>G	3 months post-transplant	90	1	8628	0.012%	90	0	10589	0.000%
AML 129	chr9:103359925T>C	3 months post-transplant	90	1	14038	0.007%	90	0	6720	0.000%
AML 130	chr1:106285578T>G	3 months post-transplant	99	1	13841	0.007%	89	0	9873	0.000%
AML 130	chr10:34621587A>T	3 months post-transplant	99	7	14921	0.047%	89	6	9455	0.063%
AML 130	chr10:85852159T>C	3 months post-transplant	99	1	10585	0.009%	89	0	11765	0.000%
AML 130	chr11:122692233A>G	3 months post-transplant	99	4	15395	0.026%	89	2	10447	0.019%
AML 130	chr11:38802150A>G	3 months post-transplant	99	1	15179	0.007%	89	0	10315	0.000%
AML 130	chr12:104741654A>G	3 months post-transplant	99	1	16767	0.006%	89	1	10985	0.009%
AML 130	chr12:13896169A>G	3 months post-transplant	99	0	8720	0.000%	89	1	10009	0.010%
AML 130	chr14:26683707T>C	3 months post-transplant	99	3	7273	0.041%	89	3	10226	0.029%
AML 130	chr14:48973412A>G	3 months post-transplant	99	1	14850	0.007%	89	0	11400	0.000%
AML 130	chr14:90423441C>T	3 months post-transplant	99	23	15336	0.150%	89	10	11317	0.088%
AML 130	chr14:98255192T>C	3 months post-transplant	99	31	19592	0.158%	89	11	11681	0.094%
AML 130	chr16:46743605A>G	3 months post-transplant	99	3	5701	0.053%	89	6	11439	0.052%
AML 130	chr16:65528696G>C	3 months post-transplant	99	14	14634	0.096%	89	9	10289	0.087%
AML 130	chr2:119166886A>C	3 months post-transplant	99	1	11954	0.008%	89	0	10450	0.000%
AML 130	chr2:202559796G>C	3 months post-transplant	99	1	13997	0.007%	89	0	7475	0.000%
AML 130	chr20:11727205C>G	3 months post-transplant	99	2	13017	0.015%	89	0	8953	0.000%
AML 130	chr3:128411745G>C	3 months post-transplant	99	1	14549	0.007%	89	0	9425	0.000%
AML 130	chr8:68116084T>C	3 months post-transplant	99	2	19360	0.010%	89	0	11859	0.000%
AML 130	chr9:78680128G>A	3 months post-transplant	99	1	18388	0.005%	89	0	9717	0.000%
AML 131	chr2:122916745A>G	3 months post-transplant	86	0	5423	0.000%	98	1	12304	0.008%
AML 235	chr1:165567242T>G	3 months post-transplant	84	1	3136	0.032%	97	0	265	0.000%
AML 235	chr1:19857444C>G	3 months post-transplant	84	4	18955	0.021%	97	0	7285	0.000%
AML 235	chr1:23429400T>C	3 months post-transplant	84	1	19771	0.005%	97	0	8192	0.000%
AML 235	chr1:25107284G>A	3 months post-transplant	84	3	20580	0.015%	97	0	8798	0.000%
AML 235	chr1:87885697C>T	3 months post-transplant	84	1	19290	0.005%	97	0	8006	0.000%
AML 235	chr10:100081931A>G	3 months post-transplant	84	4	23014	0.017%	97	0	7231	0.000%
AML 235	chr10:125533657C>T	3 months post-transplant	84	1	16837	0.006%	97	1	8066	0.012%
AML 235	chr10:17952177G>C	3 months post-transplant	84	2	21195	0.009%	97	1	8138	0.012%
AML 235	chr11:118519716>GG	3 months post-transplant	84	2	22873	0.009%	97	0	9248	0.000%

AML 235	chr11:123940800G>C	3 months post-transplant	84	2	16922	0.012%	97	1	7916	0.013%
AML 235	chr11:31249680T>C	3 months post-transplant	84	2	21673	0.009%	97	0	6895	0.000%
AML 235	chr12:221476C>T	3 months post-transplant	84	1	19926	0.005%	97	0	8979	0.000%
AML 235	chr12:25623624T>C	3 months post-transplant	84	1	13173	0.008%	97	0	2646	0.000%
AML 235	chr12:3529546T>C	3 months post-transplant	84	6	18106	0.033%	97	0	6513	0.000%
AML 235	chr12:5471503G>A	3 months post-transplant	84	1	16439	0.006%	97	0	7235	0.000%
AML 235	chr12:64733476T>C	3 months post-transplant	84	0	19483	0.000%	97	1	7103	0.014%
AML 235	chr12:96117801G>A	3 months post-transplant	84	1	22156	0.005%	97	0	8257	0.000%
AML 235	chr14:39769140G>C	3 months post-transplant	84	2	18129	0.011%	97	0	6036	0.000%
AML 235	chr14:78099338C>T	3 months post-transplant	84	1	15309	0.007%	97	0	6780	0.000%
AML 235	chr14:81676314G>A	3 months post-transplant	84	2	17255	0.012%	97	1	7200	0.014%
AML 235	chr15:31787892A>G	3 months post-transplant	84	1	9747	0.010%	97	0	2606	0.000%
AML 235	chr15:66677904G>A	3 months post-transplant	84	3	24798	0.012%	97	0	8769	0.000%
AML 235	chr15:69028002C>T	3 months post-transplant	84	4	23852	0.017%	97	0	8735	0.000%
AML 235	chr16:85603369>CC	3 months post-transplant	84	1	13648	0.007%	97	0	5493	0.000%
AML 235	chr16:87008603A>G	3 months post-transplant	84	1	18372	0.005%	97	0	7999	0.000%
AML 235	chr17:52250848A>	3 months post-transplant	84	1	17519	0.006%	97	0	5959	0.000%
AML 235	chr18:24561167G>A	3 months post-transplant	84	2	23054	0.009%	97	0	8178	0.000%
AML 235	chr18:40037787G>T	3 months post-transplant	84	3	17864	0.017%	97	0	7966	0.000%
AML 235	chr19:50383921C>G	3 months post-transplant	84	1	12834	0.008%	97	0	8559	0.000%
AML 235	chr2:7857516C>T	3 months post-transplant	84	2	24878	0.008%	97	0	9208	0.000%
AML 235	chr21:40416188G>A	3 months post-transplant	84	4	18896	0.021%	97	1	9285	0.011%
AML 235	chr22:25780119G>A	3 months post-transplant	84	2	16464	0.012%	97	0	4735	0.000%
AML 235	chr22:29698198A>G	3 months post-transplant	84	3	19397	0.015%	97	0	7999	0.000%
AML 235	chr22:29837510T>C	3 months post-transplant	84	3	16773	0.018%	97	0	7568	0.000%
AML 235	chr3:107163518T>C	3 months post-transplant	84	1	15717	0.006%	97	0	5692	0.000%
AML 235	chr3:175778314A>G	3 months post-transplant	84	2	19439	0.010%	97	0	7347	0.000%
AML 235	chr3:181066160T>C	3 months post-transplant	84	3	18649	0.016%	97	0	7156	0.000%
AML 235	chr3:50812223A>G	3 months post-transplant	84	5	17985	0.028%	97	0	8316	0.000%
AML 235	chr3:54031649G>A	3 months post-transplant	84	1	16456	0.006%	97	0	7374	0.000%
AML 235	chr5:3126570T>C	3 months post-transplant	84	2	17827	0.011%	97	0	7706	0.000%
AML 235	chr5:4306175C>T	3 months post-transplant	84	2	20906	0.010%	97	0	8503	0.000%
AML 235	chr5:45510620C>T	3 months post-transplant	84	1	17326	0.006%	97	0	7411	0.000%
AML 235	chr6:105955681A>G	3 months post-transplant	84	2	15549	0.013%	97	0	7067	0.000%
AML 235	chr6:126010195G>A	3 months post-transplant	84	2	17697	0.011%	97	0	7042	0.000%
AML 235	chr6:138865853C>G	3 months post-transplant	84	1	13057	0.008%	97	0	6081	0.000%
AML 235	chr6:167547928C>A	3 months post-transplant	84	3	21690	0.014%	97	0	8111	0.000%
AML 235	chr6:7186967T>A	3 months post-transplant	84	1	19420	0.005%	97	0	7853	0.000%
AML 235	chr6:85492862C>T	3 months post-transplant	84	2	20527	0.010%	97	0	7131	0.000%
AML 235	chr6:94066528A>T	3 months post-transplant	84	3	17053	0.018%	97	0	7910	0.000%
AML 235	chr7:24086625G>A	3 months post-transplant	84	1	13730	0.007%	97	0	7391	0.000%
AML 235	chr7:33743414C>G	3 months post-transplant	84	2	18095	0.011%	97	1	7295	0.014%
AML 235	chr7:3972928G>C	3 months post-transplant	84	6	20347	0.029%	97	0	9158	0.000%
AML 235	chr8:129629953T>G	3 months post-transplant	84	35	22485	0.156%	97	0	8284	0.000%
AML 235	chr8:20884829T>C	3 months post-transplant	84	2	17583	0.011%	97	0	8081	0.000%
AML 235	chr8:2862203C>A	3 months post-transplant	84	1	17416	0.006%	97	0	7435	0.000%
AML 235	chr8:73451557T>C	3 months post-transplant	84	1	17563	0.006%	97	0	8612	0.000%
AML 235	chr8:74066786G>A	3 months post-transplant	84	1	14380	0.007%	97	0	7597	0.000%
AML 235	chr9:109084857C>T	3 months post-transplant	84	2	18935	0.011%	97	0	5980	0.000%
AML 235	chr9:135143068T>C	3 months post-transplant	84	3	23615	0.013%	97	0	7862	0.000%

AML 235	chr9:23002947C>T	3 months post-transplant	84	4	17679	0.023%	97	0	6164	0.000%
AML 235	chrX:107140810C>G	3 months post-transplant	84	2	16758	0.012%	97	0	6922	0.000%
AML 235	chrX:148725203C>T	3 months post-transplant	84	1	20273	0.005%	97	1	8546	0.012%
AML 239	chr11:112858841G>A	3 months post-transplant	83	0	4769	0.000%	97	1	11743	0.009%
AML 239	chr6:70101478G>C	3 months post-transplant	83	1	2966	0.034%	97	1	9872	0.010%
AML 239	chr8:5679049T>A	3 months post-transplant	83	0	2875	0.000%	97	1	10475	0.010%
AML 239	chr9:9417477C>T	3 months post-transplant	83	1	3973	0.025%	97	0	10985	0.000%
AML 241	chr13:42762113T>G	3 months post-transplant	92	1	18905	0.005%	92	0	8553	0.000%
AML 241	chr8:137985582C>T	3 months post-transplant	92	1	18342	0.005%	92	0	9064	0.000%
AML 245	chr11:32396398G>	3 months post-transplant	92	1	4818	0.021%	98	0	14904	0.000%
AML 245	chr11:7763661G>A	3 months post-transplant	92	1	4084	0.024%	98	0	7661	0.000%
AML 245	chr14:95613620G>A	3 months post-transplant	92	0	4086	0.000%	98	1	12954	0.008%
AML 245	chr20:20833493G>A	3 months post-transplant	92	1	5538	0.018%	98	0	17359	0.000%
AML 245	chr4:119684667T>C	3 months post-transplant	92	0	3551	0.000%	98	1	8971	0.011%
AML 245	chr5:140005751T>C	3 months post-transplant	92	1	4795	0.021%	98	0	15289	0.000%
AML 245	chr7:156002360C>T	3 months post-transplant	92	0	4798	0.000%	98	1	13550	0.007%
AML 248	chr1:14609249G>A	3 months post-transplant	86	14	13551	0.103%	93	0	9171	0.000%
AML 248	chr1:147656369C>T	3 months post-transplant	86	29	13972	0.208%	93	0	6938	0.000%
AML 248	chr1:203737152A>G	3 months post-transplant	86	12	8374	0.143%	93	0	4345	0.000%
AML 248	chr1:2820056G>A	3 months post-transplant	86	25	15844	0.158%	93	0	6668	0.000%
AML 248	chr1:40866191T>C	3 months post-transplant	86	44	16085	0.274%	93	0	8049	0.000%
AML 248	chr1:7466894C>T	3 months post-transplant	86	21	17285	0.121%	93	0	7897	0.000%
AML 248	chr10:118507409T>C	3 months post-transplant	86	14	13005	0.108%	93	0	7468	0.000%
AML 248	chr10:16313939T>A	3 months post-transplant	86	16	11619	0.138%	93	0	8859	0.000%
AML 248	chr10:17429036>A	3 months post-transplant	86	2	8602	0.023%	93	0	8543	0.000%
AML 248	chr11:11641040G>A	3 months post-transplant	86	22	15570	0.141%	93	0	9108	0.000%
AML 248	hr11:134654301>GGGTGCAT	3 months post-transplant	86	16	13596	0.118%	93	0	8612	0.000%
AML 248	chr11:36861290G>A	3 months post-transplant	86	23	10562	0.218%	93	0	7833	0.000%
AML 248	chr11:94722188G>A	3 months post-transplant	86	16	12057	0.133%	93	0	7914	0.000%
AML 248	chr13:48117971A>G	3 months post-transplant	86	31	15740	0.197%	93	0	8454	0.000%
AML 248	chr13:62224453C>T	3 months post-transplant	86	15	11148	0.135%	93	0	8501	0.000%
AML 248	chr13:93287606G>A	3 months post-transplant	86	16	9217	0.174%	93	0	7852	0.000%
AML 248	chr14:39119285G>A	3 months post-transplant	86	19	12296	0.155%	93	0	7709	0.000%
AML 248	chr15:37780604C>T	3 months post-transplant	86	1	15622	0.006%	93	0	8598	0.000%
AML 248	chr15:85938083C>T	3 months post-transplant	86	25	14073	0.178%	93	0	8675	0.000%
AML 248	chr16:9871241>A	3 months post-transplant	86	16	10128	0.158%	93	0	8275	0.000%
AML 248	chr17:5766396T>C	3 months post-transplant	86	38	19084	0.199%	93	0	9599	0.000%
AML 248	chr19:19096041C>G	3 months post-transplant	86	21	17276	0.122%	93	0	9013	0.000%
AML 248	chr19:33701142C>T	3 months post-transplant	86	22	18540	0.119%	93	0	8866	0.000%
AML 248	chr2:158126080G>A	3 months post-transplant	86	16	12134	0.132%	93	0	8069	0.000%
AML 248	chr2:18197156G>A	3 months post-transplant	86	24	9890	0.243%	93	0	6906	0.000%
AML 248	chr2:221271014A>T	3 months post-transplant	86	10	11033	0.091%	93	1	8708	0.011%
AML 248	chr2:239881851A>G	3 months post-transplant	86	24	15443	0.155%	93	0	8978	0.000%
AML 248	chr2:3974847C>T	3 months post-transplant	86	21	14270	0.147%	93	0	7797	0.000%
AML 248	chr2:49176542T>C	3 months post-transplant	86	18	13116	0.137%	93	0	8242	0.000%
AML 248	chr2:66358307C>T	3 months post-transplant	86	21	10289	0.204%	93	0	6181	0.000%
AML 248	chr2:9414541C>T	3 months post-transplant	86	20	14236	0.140%	93	0	8195	0.000%
AML 248	chr21:25343636C>T	3 months post-transplant	86	21	12399	0.169%	93	0	8442	0.000%
AML 248	chr21:37677112C>T	3 months post-transplant	86	19	14567	0.130%	93	0	8261	0.000%
AML 248	chr22:33909039C>T	3 months post-transplant	86	23	13901	0.165%	93	0	7693	0.000%

AML 248	chr22:47378266G>A	3 months post-transplant	86	17	13219	0.129%	93	0	7197	0.000%
AML 248	chr3:105395780G>C	3 months post-transplant	86	27	10508	0.257%	93	0	7937	0.000%
AML 248	chr3:138365619T>C	3 months post-transplant	86	24	13650	0.176%	93	0	8357	0.000%
AML 248	chr3:15268647T>C	3 months post-transplant	86	37	16890	0.219%	93	0	8741	0.000%
AML 248	chr3:155069427G>T	3 months post-transplant	86	31	13335	0.232%	93	0	8622	0.000%
AML 248	chr3:193344044G>T	3 months post-transplant	86	21	15133	0.139%	93	0	8790	0.000%
AML 248	chr3:36719449C>T	3 months post-transplant	86	23	12948	0.178%	93	0	8235	0.000%
AML 248	chr4:114087248T>C	3 months post-transplant	86	7	3246	0.216%	93	0	2695	0.000%
AML 248	chr5:138420903C>T	3 months post-transplant	86	33	15156	0.218%	93	0	8175	0.000%
AML 248	chr5:37768102G>C	3 months post-transplant	86	20	15150	0.132%	93	0	8904	0.000%
AML 248	chr5:38936499G>	3 months post-transplant	86	22	13480	0.163%	93	0	9087	0.000%
AML 248	chr5:5942386C>T	3 months post-transplant	86	19	12686	0.150%	93	0	9028	0.000%
AML 248	chr6:109374931A>G	3 months post-transplant	86	2	9685	0.021%	93	0	6685	0.000%
AML 248	chr6:84798255C>A	3 months post-transplant	86	30	17876	0.168%	93	0	9091	0.000%
AML 248	chr7:118695138C>T	3 months post-transplant	86	25	10995	0.227%	93	0	7033	0.000%
AML 248	chr7:132095315C>T	3 months post-transplant	86	15	12444	0.121%	93	0	6710	0.000%
AML 248	chr7:132300844G>A	3 months post-transplant	86	22	14325	0.154%	93	0	8123	0.000%
AML 248	chr7:158236471C>T	3 months post-transplant	86	33	17353	0.190%	93	0	7305	0.000%
AML 248	chr7:8418476G>A	3 months post-transplant	86	40	10804	0.370%	93	0	7294	0.000%
AML 248	chr8:139131792G>A	3 months post-transplant	86	14	9241	0.151%	93	0	8022	0.000%
AML 248	chr8:32518458T>C	3 months post-transplant	86	16	10759	0.149%	93	0	7591	0.000%
AML 248	chr8:3460641T>C	3 months post-transplant	86	15	9912	0.151%	93	0	6154	0.000%
AML 248	chr8:41719460G>A	3 months post-transplant	86	27	16085	0.168%	93	0	7068	0.000%
AML 248	chr8:60062081G>C	3 months post-transplant	86	17	12676	0.134%	93	0	7674	0.000%
AML 248	chr9:132157050C>T	3 months post-transplant	86	19	14354	0.132%	93	0	7673	0.000%
AML 248	chrX:17613357G>A	3 months post-transplant	86	1	9019	0.011%	93	0	4637	0.000%
AML 251	chr1:155904480T>A	3 months post-transplant	87	1	33055	0.003%	81	0	4945	0.000%
AML 251	chr1:179669423G>C	3 months post-transplant	87	2	41948	0.005%	81	0	5275	0.000%
AML 251	chr1:223462447G>C	3 months post-transplant	87	2	30328	0.007%	81	0	4764	0.000%
AML 251	chr1:75025278C>G	3 months post-transplant	87	3	29815	0.010%	81	0	3176	0.000%
AML 251	chr10:106665258G>C	3 months post-transplant	87	2	42435	0.005%	81	0	4075	0.000%
AML 251	chr10:36621510G>C	3 months post-transplant	87	1	30853	0.003%	81	0	3412	0.000%
AML 251	chr11:123643117C>G	3 months post-transplant	87	1	33854	0.003%	81	0	5771	0.000%
AML 251	chr11:5515469G>C	3 months post-transplant	87	0	41721	0.000%	81	1	5416	0.018%
AML 251	chr11:81220644G>A	3 months post-transplant	87	4	30807	0.013%	81	0	4254	0.000%
AML 251	chr11:99020392T>C	3 months post-transplant	87	1	34651	0.003%	81	0	4423	0.000%
AML 251	chr12:115262331G>	3 months post-transplant	87	1	44373	0.002%	81	0	4967	0.000%
AML 251	chr12:49052371T>C	3 months post-transplant	87	7	37299	0.019%	81	0	6322	0.000%
AML 251	chr12:85898023T>C	3 months post-transplant	87	3	31220	0.010%	81	0	4217	0.000%
AML 251	chr12:93574960>GA	3 months post-transplant	87	2	36510	0.005%	81	0	4330	0.000%
AML 251	chr12:97751063G>A	3 months post-transplant	87	3	30411	0.010%	81	0	4106	0.000%
AML 251	chr13:65393072G>T	3 months post-transplant	87	1	31220	0.003%	81	0	3586	0.000%
AML 251	chr13:85906764G>T	3 months post-transplant	87	3	33022	0.009%	81	0	3279	0.000%
AML 251	chr13:96373712C>T	3 months post-transplant	87	2	32389	0.006%	81	0	3827	0.000%
AML 251	chr14:29615725G>C	3 months post-transplant	87	1	32112	0.003%	81	0	3560	0.000%
AML 251	chr15:101389694T>C	3 months post-transplant	87	1	40168	0.002%	81	0	6074	0.000%
AML 251	chr17:30203376A>G	3 months post-transplant	87	1	34021	0.003%	81	0	4725	0.000%
AML 251	chr18:54001774G>C	3 months post-transplant	87	2	40419	0.005%	81	0	4731	0.000%
AML 251	chr18:60227705C>T	3 months post-transplant	87	2	35395	0.006%	81	0	4053	0.000%
AML 251	chr18:70524437A>T	3 months post-transplant	87	1	42581	0.002%	81	0	4599	0.000%

AML 251	chr19:33623896C>A	3 months post-transplant	87	1	36554	0.003%	81	0	5847	0.000%
AML 251	chr2:212491338A>G	3 months post-transplant	87	3	30521	0.010%	81	0	3172	0.000%
AML 251	chr2:239529022C>T	3 months post-transplant	87	1	37809	0.003%	81	0	6012	0.000%
AML 251	chr2:30056856G>A	3 months post-transplant	87	1	38941	0.003%	81	0	4313	0.000%
AML 251	chr21:46412475C>T	3 months post-transplant	87	3	36178	0.008%	81	0	5949	0.000%
AML 251	chr3:181814957G>A	3 months post-transplant	87	1	32875	0.003%	81	0	4103	0.000%
AML 251	chr3:193785856G>C	3 months post-transplant	87	2	37546	0.005%	81	0	5810	0.000%
AML 251	chr3:48782120T>G	3 months post-transplant	87	2	34866	0.006%	81	0	4759	0.000%
AML 251	chr4:4944193G>T	3 months post-transplant	87	2	31513	0.006%	81	1	6202	0.016%
AML 251	chr5:9783059G>A	3 months post-transplant	87	1	37040	0.003%	81	0	4917	0.000%
AML 251	chr6:163301683T>C	3 months post-transplant	87	2	40580	0.005%	81	0	4989	0.000%
AML 251	chr6:163336041>GGGGCA	3 months post-transplant	87	3	34762	0.009%	81	0	5511	0.000%
AML 251	chr6:68956057G>C	3 months post-transplant	87	1	38515	0.003%	81	0	4790	0.000%
AML 251	chr7:100561558C>G	3 months post-transplant	87	1	44329	0.002%	81	0	6333	0.000%
AML 251	chr7:117041084G>C	3 months post-transplant	87	1	28745	0.003%	81	0	3183	0.000%
AML 251	chr7:147509396T>C	3 months post-transplant	87	2	36020	0.006%	81	0	3954	0.000%
AML 251	chr7:47605953>TGCCC	3 months post-transplant	87	3	44147	0.007%	81	0	5598	0.000%
AML 251	chr8:102015064T>C	3 months post-transplant	87	3	42190	0.007%	81	0	4695	0.000%
AML 251	chr8:14238270C>A	3 months post-transplant	87	4	31014	0.013%	81	0	3369	0.000%
AML 251	chr9:137746715C>T	3 months post-transplant	87	2	28570	0.007%	81	0	4556	0.000%
AML 251	chr9:5231567T>A	3 months post-transplant	87	3	41691	0.007%	81	0	6388	0.000%
AML 251	chrX:29072621>CCCC	3 months post-transplant	87	2	20966	0.010%	81	0	1832	0.000%

Mutation genomic coordinates refer to hg38

Table S4: Driver mutations identified.

Patient	Chromosome	Position	Base from	Base to	Gene and Amino Acid Change	Included in v96 panel?
AML 121	chr9	5073770	G	T	JAK2 p.V617F	No
AML 122	chr2	208248388	C	T	IDH1 p.R132H	Yes
AML 122	chr1	114716123	C	T	NRAS p.G13D	Yes
AML 122	chr13	28028192	G	A	FLT3 p.A680V	No
AML 123	chr15	90088702	C	T	IDH2 p.R140Q	Yes
AML 123	chr5	171410539	-	TCTG	NPM1 p.W288fs	Yes
AML 123	chr13	28034116	-	GAGATCATATTCATATTCTCTGAAATCAACG	FLT3 ITD	No
AML 124	chr17	31337858	C	T	NF1 p.Q2207*	No
AML 124	chr1	114713909	G	T	NRAS p.Q61K	No
AML 124	chr1	114716123	C	T	NRAS p.G13D	No
AML 125	chr5	171410539	-	TCTG	NPM1 p.W288fs	Yes
AML 126	chr2	25234373	C	T	DNMT3A p.R882H	Yes
AML 127	chr5	171410541	-	TGCA	NPM1 p.W288fs	Yes
AML 127	chr13	28034125, 28034132	-	TCATATTCTCTGAAATCAACGTAGAAGTACTCATTATC TGAGGAGCCGGTCACCTG, TCTCTGAAATCAACGTAG	FLT3 ITD	No
AML 128	chr2	25245303	-	A	DNMT3A p.V502f	No
AML 128	chr2	208248389	G	A	IDH1 p.R132C	Yes
AML 128	chr13	28034126	-	TCATATTCTCTGAAATCAACGTAG	FLT3 ITD	No
AML 129	chr21	43094670	C	T	U2AF1 p.R156H	No
AML 129	chr13	28034100	-	TTGGAAACTCCCATTTGAGATCATGGGGGTCCATCT	FLT3 ITD	No
AML 130	chr3	128483911	-	TA	GATA2 p.H323fs	Yes
AML 131	chr9	5073770	G	T	JAK2 p.V617F	No
AML 132	chr17	7676011	T	C	TP53 p.K120E	Yes
AML 133	chr21	34880580	C	T	RUNX1 p.R162K	Yes
AML 133	chr20	32434981	CAGCCATG	-	ASXL1 p.Q757fs	Yes
AML 133	chr2	197402981	C	T	SF3B1 p.E592K	Yes
AML 133	chr1	114713908	T	C	NRAS p.Q61R	No
AML 134	chr1	43339489	T	C	MPL p.S204P	Yes
AML 235	chr4	54733154	G	C	KIT p.D816H	No
AML 235	chr4	54733155	A	T	KIT p.D816V	No
AML 235	chr11	118519716	-	GG	KMT2A p.F3750fs	No
AML 236	chr17	7675161	G	A	TP53 p.P151S	No
AML 237	chr5	171410539	-	TCTG	NPM1 p.W288fs	No
AML 237	chr15	90088702	C	T	IDH2 p.R140Q	No
AML 238	chr1	114713908	T	C	NRAS p.Q61R	No
AML 239	chr2	25234373	C	T	DNMT3A p.R882H	No
AML 239	chr5	171410539	-	TCTG	NPM1 p.W288fs	No
AML 239	chr13	28034099	-	TGGAAACTCCCATTTGAGATCATATTCATATTCTCTGA AATCAACGTAGAAGTACTCAT	FLT3 ITD	No
AML 240	chr1	114716126	C	T	NRAS p.G12D	Yes
AML 240	chr5	171410540	-	CTGC	NPM1 p.W288fs	No
AML 241	chr11	108329172	A	C	ATM p.Q2414P	No
AML 242	chr1	114716123	C	T	NRAS p.G13D	No
AML 242	chr1	114716124	C	G	NRAS p.G13R	No
AML 242	chr17	76736877	G	T	SRSF2 p.P95H	No
AML 242	chr20	32434638	-	G	ASXL1 p.G646fs	No
AML 242	chr21	34880642	-	GAGAAATAAAGAAGAGTTT	RUNX1 p.A142fs	No
AML 242	chrX	40057168	G	A	BCOR p.Q1494*	No
AML 242	chrX	124066193	-	T	STAG2 p.W706S	No
AML 244	chr5	171410541	-	TGCA	NPM1 p.W288fs	No
AML 244	chr12	25245347	C	T	KRAS p.G13D	Yes
AML 244	chr13	28018486	T	A	FLT3 p.N841I	No
AML 245	chr5	171410539	-	TCTG	NPM1 p.W288fs	No
AML 245	chr11	32396398	G	-	WT1 p.R370fs	Yes
AML 245	chr13	28034090	-	AGTGGCGGAATTTTCTCTTGGAAACTCCCATTTGAGAT CATATTCATATTCTCTGAAATCAACGTAGAAGTACTCA TTATCTGAAGTGGCGG	FLT3 ITD	No
AML 245	chr13	28034143	AA	TG	FLT3 p.V592A	No
AML 246	chr17	7675124	T	C	TP53 p.Y163C	Yes
AML 247	chr1	114713907	T	G	NRAS p.Q61H	No
AML 247	chr1	114713908	T	A	NRAS p.Q61L	No
AML 247	chr1	114716123	C	T	NRAS p.G13D	Yes
AML 247	chr1	114716126	C	G	NRAS p.G12A	No
AML 247	chr12	25245351	C	A	KRAS p.G12C	No
AML 247	chr13	28018505	C	A	FLT3 p.D835Y	No
AML 248	chr4	105272684	C	T	TET2 p.Q1435*	No
AML 249	chr2	197402635	C	G	SF3B1 p.K666N	No
AML 249	chr17	76736877	G	A	SRSF2 p.P95L	No
AML 249	chr21	34880580	C	T	RUNX1 p.R162K	No

AML 249	chr21	34886952	-	C	RUNX1 p.V81fs	No
AML 250	chr5	171410539	-	TCTG	NPM1 p.W288fs	No
AML 250	chr13	28034123	-	TATTCATATTCTCTGAAATCA	FLT3 ITD	No
AML 250	chr15	90088702	C	A	IDH2 p.R140L	No
AML 251	chr11	118490196	A	G	KMT2A Pp.Q1548R	No
AML 251	chr12	49052371	T	C	KMT2D p.M438V	Yes
AML 251	chr15	90088702	C	T	IDH2 p.R140Q	No

Mutation genomic coordinates refer to hg38
